# The contribution of open comments to understanding the results from the Hospital Survey on Patient Safety Culture (HSOPS): A qualitative study

**DOI:** 10.1371/journal.pone.0196089

**Published:** 2018-04-19

**Authors:** Bastien Boussat, Kevin Kamalanavin, Patrice François

**Affiliations:** 1 Quality of care unit, Grenoble University Hospital, Grenoble, France; 2 TIMC UMR 5525 CNRS, Université Grenoble Alpes, Grenoble, France; 3 Department of political science, Institute of Political Studies, Grenoble, France; TNO, NETHERLANDS

## Abstract

**Introduction:**

To develop high-quality and safe healthcare, a good safety culture is an important feature of healthcare-providing structures. The objective of this study was to analyze the qualitative data of the comments section of a Hospital Survey on Patient Safety (HSOPS) questionnaire to clarify the answers given to the closed questions.

**Method:**

Using the original data from a cross-sectional survey of 5,064 employees at a single university hospital in France, we conducted a qualitative study by analyzing the comments of a HSOPS survey and conducting in-depth interviews with 19 healthcare providers. We submitted the comments and the interviews to a thematic analysis.

**Results:**

A total of 3,978 questionnaires were returned, with 247 comments collected. The qualitative analysis identified several structural failures. The main categories of the open comments were concordant with the lowest dimension scores found in the quantitative analysis. The most frequently reported failures were related to the staffing and hospital management support dimensions. The healthcare professionals perceived the lack of resources, including understaffing, as the major barrier to the development of a patient safety culture. Concrete organizational issues related to hospital handoffs and risk coordination were identified, such as transfers from the emergency departments and the lack of feedback following self-reporting of incidents.

**Conclusion:**

The analysis of the open comments complemented the HSOPS scores, increasing the level of detail in the description of the hospital’s patient safety culture. Combined with a classical quantitative approach used in HSOPS-based surveys, the qualitative analysis of open comments is useful to identify organizational weaknesses within the hospital.

## Introduction

Medical errors cause an estimated 251,454 deaths every year in the United States, the third leading cause of death[1], even though patient safety has been a priority for health systems for more than 15 years. In 2004, the World Health Organization created the World Alliance for Patient Safety. This alliance has developed several initiatives, programs, and guides to enhance patient safety and reduce the occurrence of medical errors.[2–4] This raises the suspicion that safety enhancement efforts may have been inhibited by structural causes.

Inspired by the experience of high-reliability organizations such as air transport and the nuclear industry,[5] safety culture is defined as “the product of individual and group values, attitudes, perceptions, competencies, and patterns of behaviour that determine the commitment to, and the style and proficiency of, an organisation’s health and safety management.”[6] Safety culture has become increasingly important, inciting both professionals and healthcare facilities to adopt behaviors and management tools promoting patient safety. The rising importance of safety culture has resulted in the need to assess it and measure it.

Among the several tools developed to assess the patient safety culture, one of the most widely used is the Hospital Survey on Patient Safety Culture (HSOPS). Created by the Agency for Healthcare Research and Quality (AHRQ), this survey consists of a self-administered questionnaire including 42 items formulated as closed questions and used to calculate composite scores for 12 dimensions of safety culture.[6,7] It also includes a comments section, but to our knowledge, few surveys using the HSOPS questionnaire have analyzed these comments.

Closed questions allow collecting standardized information that can be statistically exploited, but the information is consequently poorer. However, comments give more detailed information about the concerns of staff members. Furthermore, interviews with staff members and the qualitative method enable us to understand the logic of their behavior and the influence of work conditions on this behavior. Therefore, it may complement the answers to the questionnaire’s closed questions, thus clarifying their meaning. It also provides further information and helps identify the staff’s preoccupations and structural issues that have not been diagnosed by the survey.[8,9]

The main objective of this study was to analyze the qualitative data of the comments section of the HSOPS to refine the survey results. These data were produced by analyzing the comments collected during the HSOPS survey and the interviews conducted with front-line staff members.

## Methods

### Study design

This study examined a qualitative part of an HSOPS-based survey. We first exploited the comments left in the comments area of the HSOPS questionnaire. This analysis was used to prepare the interview guide of an interview-based qualitative study conducted to further the analysis. The institutional review board at Grenoble University Hospital (IRB 6705) reviewed the study protocol and waived the need for informed participant consent. The study protocol was approved by the Advisory Board on Medical Research Data Processing (CCTIRS) and authorization by the French Personal Data Protection Authority (CNIL) was obtained before data processing started.

### Study site

The study was conducted at a single university-affiliated hospital with a capacity of 1836 beds (including 1175 acute care beds and 661 long-term or subacute care beds), serving a predominantly urban population of 675,000 inhabitants in France. The study site reported 135,999 stays in 2014. The hospital staff comprised 4,422 registered paramedical staff and 642 board-certified physicians. The number of beds and the paramedical staff rate was similar to other French university-affiliated hospitals (2336 versus 1836 beds, and 2.4 versus 2.6 paramedical staff per bed) [10].

### Population

The HSOPS was conducted anonymously on a volunteer basis, department by department, between April 2013 and September 2014. Eligible participants were full-time or part-time (half-time or more) employees with at least 6 months of employment in the clinical, laboratory/pathology, radiology, or pharmacy departments. In accordance with the HSOPS guide,[6] this study sample encompassed clinical and nonclinical staff who had direct contact or interaction with patients and hospital staff who might not have direct contact with patients but whose work directly affected patient care.

As recommended,[6] HSOPS questionnaires were secondarily excluded if the respondent did not complete at least one survey section, answered fewer than half of the items, or answered every item with the same non-neutral response.

The comments were collected in the specific field at the end of the questionnaires. Then exclusion criteria were applied separately for the comments: illegible or incomprehensible comments and nonpertinent comments (such as “No,” “Nothing to report,” “None,” etc.) were excluded.

The interviews were conducted with 19 medical and paramedical professionals from May to July 2016. We limited the eligibility to selected categories of professionals in several selected departments. We interviewed physicians and pharmacists, head nurses, nurses, nursing aides, and stretcher bearers.

### Data collection

The questionnaires were distributed in all departments by an investigator, cooperatively with head nurses, who established the list of staff members to be included.

### Comments

Comments were collected in the comments section of the HSOPS questionnaire. They were scripted and submitted to a triple reading before being analyzed.

### Interviews

The interviews were conducted from May to July 2016 and lasted an average 40 min. Nineteen staff members were interviewed and the interviews were recorded and transcribed.

Following the analysis of the comments, we developed an interview guide to conduct semi-directive interviews with staff members. The interview guide was composed of the topics identified by the comment analysis. The interviewer agreed with the interviewees that recordings and transcriptions would not be disseminated.

The interviews were based on a thematic guide comprising one main question–“What can you tell me about patient safety in the hospital and about the main factors influencing it?”–and the topics identified by analyzing the comments. At first, the staff members interviewed were asked to speak freely on this question, raising the topics of their choice. When the staff members did not raise the topics on the guide, the interviewer suggested topics. Depending on the responses, the interviewer was free to ask the interviewee to give details, clarify, or complete the responses. The interviewer also tailored the questions to the interview’s context and to the staff member being interviewed.

### Analysis

#### Comments analysis

To analyze the comments, three interviewers conducted a thematic analysis independently and sorted the comments by topic. They then compared their results and agreed on a classification of the topics. Some comments concerned several topics and subsequently had several occurrences within different topics. Once this first sorting had been completed, the surveyors attributed one or several key words to each comment within a topic.

#### Interview analysis

We conducted an inductive qualitative textual analysis. Topic categorization of the comments analysis was used and interviews were cut into extracts, which we categorized within these topics. Then we attributed one or several key words to the extract depending on more specific topics or subjects discussed by the interviewee in this extract.

#### Statistical analysi

Background respondent characteristics and open comments were reported as numbers and percentages. We computed the individual means across three of the four items in a dimension to obtain the HSOPS dimension scores (range, 1–5). [11] Then we compared the HSOPS mean composite score values across subgroups of respondents defined by the presence or absence of an open comment. The differences between score means were tested using the Student *t*-test. In addition, we quantified the mean differences using the Cohen *d* effect size. Two-sided *P*-values lower than 0.05 were considered statistically significant. All analyses were performed using Stata Version 14.0 (Stata Corporation, College Station, TX, USA).

## Results

**Fig 1:**
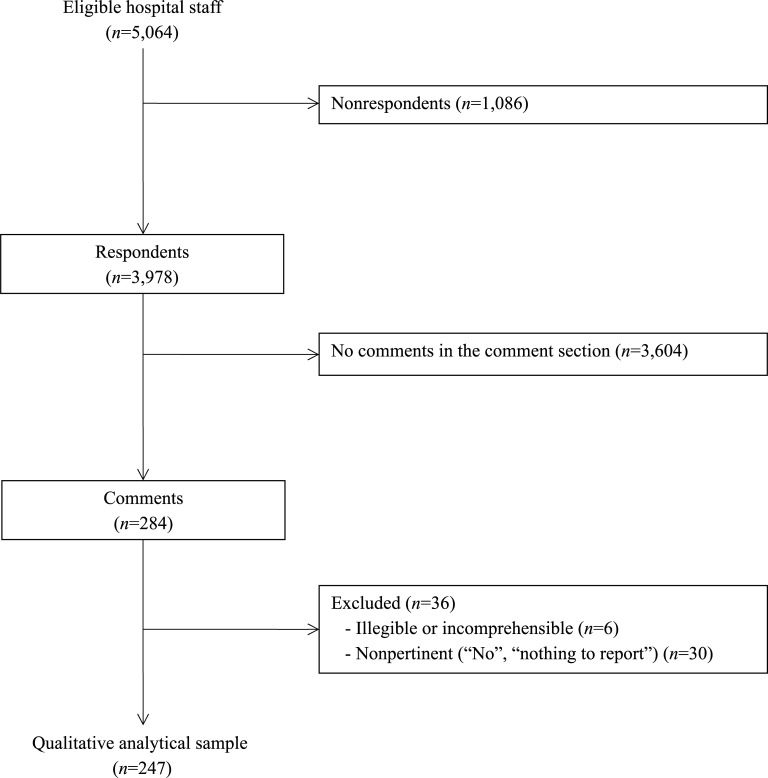
Participation and comments in the HSOPS survey.

The response rate to the HSOPS survey was 78.6% (*n* = 3,978). Among the questionnaires returned, 284 had text in the comments area; 36 questionnaires were excluded according to our exclusion criteria (Fig 1).

The majority of the respondents (80.6% for the HSOPS survey, 87.5% for the comments, and 68.4% for the interviews) were women. In both the returned questionnaires and the comments, the majority of respondents were under 46 years of age (63.2% for the HSOPS, 60.9% for the comments). The subject’s age was not requested during the interviews (Table 1).

**Table 1:** Respondent characteristics.

	HSOPS		Analyzable comment		Interviews	
Characteristics* *N* (%)	*n =* 3978		*n =* 247		*n =* 19	
Female	3,016	(80.6)	209	(87.5)	13	(68.4)
Age class (years)						
Up to 35	1,406	(37.5)	82	(34.2)		
36–45	966	(25.7)	64	(26.7)		
46–55	967	(25.7)	66	(27.5)		
56 or older	415	(11.1)	28	(11.7)		
Occupational group						
Head nurse and nurse	1,386	(36.3)	94	(38.4)	9	(47.4)
Nursing assistant	708	(18.6)	61	(24.9)	1	(5.3)
Physician	436	(11.4)	17	(6.9)	7	(36.8)
Other healthcare provider	124	(3.3)	8	(3.3)	2	(10.5)
Administrative	331	(8.7)	33	(13.5)	0	(0)
Technical	378	(9.7)	3	(1.2)	0	(0)
Other	450	(11.8)	29	(11.8)	0	(0)

* Values were missing for gender (HSOPS, *n =* 147; Analyzable comment, *n* = 8); age (HSOPS, *n* = 224; Analyzable comment, *n* = 7); and occupational group (HSOPS, *n* = 75; Analyzable comment, *n =* 2).

### HSOPS dimension scores

The mean dimension scores ranged from 2.67 for the dimension with the lowest score (hospital management support) to 3.54 positive answers for the dimension with the highest score (teamwork within hospital units) (Table 2). The two other dimensions with unfavorable mean scores concerned “staffing” and “hospital handoffs & transitions” (2.88).

**Table 2:** Results of the HSOPS.

Dimension	Score	Rank
D1: Overall perceptions of safety	3.21	7
D2: Frequency of event reporting	3.38	5
D3: Supervisor expectations & actions	3.50	2
D4: Organizational learning	3.40	4
D5: Teamwork within hospital units	3.54	1
D6: Communication openness	3.50	2
D7: Feedback and communication about error	3.38	5
D8: Nonpunitive response to error	2.94	9
D9: Staffing	2.88	10
D10: Hospital management support	2.67	12
D11: Teamwork across hospital units	3.05	8
D12: Hospital handoffs & transitions	2.88	10

**Fig 2:**
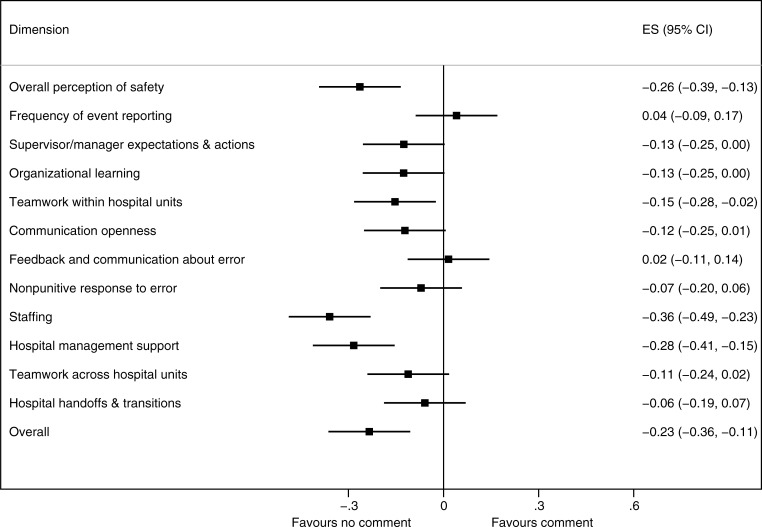
Comparison of HSOPS scores according to the presence of open comments (effect size).

Fig 2 shows the association between the presence of an open comment and the patient safety culture dimensions scores. Compared with the absence of a comment, the presence of a comment was associated with a statistically significant lower patient safety culture for four of the 12 HSOPS dimensions (overall effect size, −0.23; 95% confidence interval, −0.36 to −0.10, *P*<0.001). The three greatest differences in HSOPS score were related to “overall perception of safety,” “staffing,” and “hospital management support”.

### Classification of comments

The 247 analyzable comments were independently classified by three surveyors, who then agreed on the following classification: questionnaire (101 occurrences), staffing and hospital management support (98), organization (41), adverse event reporting and risk management support (25), premises and equipment (16) and staff safety (5). (Table 3)

**Table 3:** Categorization of comments (*n* = 247) by topics (*n* = 286) and key words.

Subject	N	(%)	Key words (occurrence)
Questionnaire	101	(35)	Unsuitable questionnaire (51) Complementary information on an answer (17) Worries about anonymity (9) Questions about the objectives of the survey (8) Problems responding (7) About the questionnaire: satisfaction (5) dissatisfaction (4)
Staffing and hospital management support	97	(34)	Fatigue (16) and stress (11) including exhaustion (5) and professional distress (1) Difficulty replacing missing staff (12) interim staff (6) Time and speed imperatives (7) Hierarchy (4) including positive support (1) lack of trust and support (3) Staff concerned by patient safety (3) Problems with overtime (3) Difficulty taking time off (3) Difficulties/lack of time to participate in safety organizations or training (3)
Organization	42	(15)	Cooperation/coordination between departments (10) Transmission/communication (10) Teamwork (9): lack (6) or appropriate (3) Beds in the corridor (3) Patient transfer (2) from emergency unit (1) or to diagnostic imaging department (1) Redeployment of staff (2) Lack of communication on hospital’s actions (2)
Adverse event reporting and risk management	25	(9)	Reporting (13) including report not taken into consideration (7) no feedback on reporting (4) lack of time (3) fear of reporting (3) Involvement in patient safety management system (9), including Morbidity and Mortality Conference (4) and Experience Feedback Committee (5) Blame culture (3)
Premises / equipment	16	(6)	Premises (5) including Patient comfort (5) risks for the patient (3) patient privacy (2) hygiene (1) Equipment (13) including computer system (5) lack of equipment (4) inadequate or defective equipment (3)
Staff safety	5	(2)	Violent patient (3) Risks related to equipment (1) Risks related to night work (1)

### Results of analysis of comments and interviews

#### Questionnaire and survey

One hundred one comments raised issues about the questionnaire or the survey. Most of these comments declared the questionnaire to be unsuitable. Some of them explained the questionnaire was unsuitable for their occupational group (particularly for secretaries). Other comments considered it to be unsuitable to evaluate patient safety. Seven respondents had difficulties answering one or several items. Seventeen respondents used the comments to give complementary information about an answer. Eight respondents had questions about the objective of the survey and nine had worries about anonymity. We did not investigate this topic further in the interviews.

#### Staffing and hospital management support: “Chronic understaffing in the department seriously affects patient safety” (Comment)

Staffing was a frequently raised topic in the comments: all comments in this topic complained about understaffing and heavy workload. Comments indicated that this understaffing was responsible for fatigue, stress, and a decrease in patient safety. Comments also reported problems stemming from missing staff (e.g., sick leave). The interviews confirmed the problem of understaffing, which was nearly always mentioned. Interviewees from all occupational groups reported understaffing and the resulting problems (stress, fatigue, absenteeism), linking it with a decrease in quality and safety in patient care.

Several professionals interviewed clearly and spontaneously talked about professional distress and even discontent stemming from a desire to resigning from their job or from seeing colleagues resign, which led to a problem of skill preservation within teams. Some professionals declared feeling that their work no longer had meaning for them. Even more interviewees declared feeling dissatisfied with their work: they considered that the work conditions made it impossible to give patients proper care.

“I don’t say it’s systematic but sometimes the working conditions affect the quality of the care we provide. I know that some colleagues have quit their job because of this, because they couldn’t offer an acceptable quality of care and they preferred to leave and transfer to another hospital that allowed them to provide a better quality of care” (Physician 3).

Some interviewees declared the problem of skill preservation and staff departure was significant in their department, with nearly 20% of a team leaving the hospital per year: “in a team of 58 people, 10 to 12 people quit every year” (Head nurse 5). The difficulty of replacing missing staff worsened the understaffing problem.

The staff members leaving comments on the questionnaires reported the risks associated with lack of adequate time. They also explained that management seemed more concerned with budgetary objectives than quality and safety. One comment explained that “The hospital managers have some good ideas, such as the implementation of training courses. But once again, understaffing shows that safety is not really a priority” (Comment). These results prompted us to investigate this issue further in the interviews.

During the interviews, staff members mentioned problems with management, explaining that management had contradictory objectives and preferred to take budgetary and economic objectives into consideration rather than quality and safety. Moreover, several professionals reported that there was a lack of consideration, respect (especially in emails), and listening on the part of management. More generally, the interviewees underlined a lack of resources in the hospital. The management was also said to be cut off from the realities of the workplace.

“It is a daily struggle against management: they want to reduce our resources while asking us to increase activity” (Physician 2).

This increased activity without increased resources resulted in problematic situations. Some staff members explained that the availability of beds did not always allow transferring a patient to the appropriate department. The interviewees explained that in these situations they did their best for the patients but admitted they did not have the skills required to give them appropriate care. The lack of available beds led to installing additional beds in the corridors. Corridor beds were reported to be a significant problem and to create significant risks since this practice increased the workload of all staff members and equipment using electrical power could not be used for these beds.

#### Organization and cooperation: “Better communication between departments would significantly reduce adverse events” (Comment)

Communication and organizational issues were often reported in the comments, mainly concerning transmission of information and transferring patients. The interviews confirmed that handoffs and transitions in care were sometimes difficult. They pointed out that information was sometimes incomplete. There was also a problem of communication within departments. Interviewees explained that information transmission and communication was well organized by protocols, but they underlined that this was not always the case.

“We organized and protocoled communication and patient transfer because things are very difficult if this is not done” (Physician 5).

Transfers from the emergency department were sometimes made without reevaluation of the patient’s condition. Beds in the corridors, partly due to lack of bed availability and resources, were also the consequence of a lack of cooperation between the receiving department and the emergency department. Interviewees conceded that time constraints did not always allow correctly transmitting information about the patient, especially in a department such as the emergency department where patient influx was not predictable.

“When patients come from the emergency department, they are not always reevaluated before their transfer… And sometimes we are surprised because the patients are not like we have been told because their condition has evolved…” (Physician 4).

#### Adverse event reporting and risk management: “We stopped reporting adverse events because we feel that there is no feedback.” (Comment)

Several comments mentioned issues related to adverse event reporting. The two most frequently cited problems were the lack of feedback following self-reporting and the lack of time to report. Other healthcare professionals declared a fear of punishment related to medical error, resulting in underreporting serious adverse events. Interestingly, adverse event reporting was considered as a source of interprofessional conflict:

“In reporting incidents, we could be more tactful, respectful and discreet towards one another and make decisions within nonviolent communication” (Comment).

“Adverse event reporting is too time-consuming and mistakes don’t receive the attention they deserve” (Comment).

Some professionals indicated they were involved in risk management activities and related this kind of activity to a good patient safety culture. They cited two programs designed to involve the medical team in patient safety management: experience feedback committees (EFC) and morbidity and mortality conferences (MMC):

“In this department, many risk management programs have been set up: EFCs and MMCs”. (Comment).

“Patient safety, EFCs, and quality procedures have been developed and implemented satisfactorily in my department” (Comment).

On the other hand, a nurse regretted that nurses were not allowed the time to participate in EFC or MMC meetings:

“For a nurse, it is unfortunately impossible to take the time to participate in EFC or MMC activities” (Nurse 2).

#### Equipment: “The equipment is increasingly fragile and ill-suited.” (Comment)

Several comments reported different equipment problems.

*“*The equipment is increasingly fragile and ill-suited. A recent example in the department is a perfusion line that spontaneously broke, fortunately before it was set up on the patient” (Comment).

These issues were also raised in the interviews. They concerned the quality of the equipment, the quantity of equipment, and ill-suited equipment. Staff members also complained about repair delays. Interviewees felt the choice of the equipment was made based on economic criteria and not quality or suitability.

“It’s a problem when the equipment you are working with and are accustomed to is replaced with something worse, because most of the time when equipment is changed it is replaced with something of lower quality. This is really a disgrace” (Nurse 1).

The employees interviewed declared that these problems involve a real risk for the patient. It was also explained that even when it does not put the patient at risk it deteriorates the quality of the care and comfort for both patient and staff.

#### Staff safety

Staff safety issues were raised by nine comments and in some of the interviews. The main risks were musculoskeletal injuries, needle stick and blood exposure, and finally a risk of violence in some departments, notably the emergency department.

#### Thematic analysis stratified by occupational group

The stratified analysis confirmed that most administrative agents (80%) commented on the unsuitability of the HSOPS for their professional group (Table 4). The other occupational groups commented more often within the “Staffing and hospital support” topic. The highest proportion of comments related to this topic was found for nursing assistants (52%) and nurses (41%). Concern for staffing and management support was also shared by physicians, but in a lower proportion (32%). Finally, the topic “adverse event reporting and risk management” was also a primary concern for the physicians participating in the survey (28%).

**Table 4:** Classification of comments by topic, stratified by occupational group.

Occupational group*, N (%)	Questionnaire		Staffing and hospital management support		Organization		Adverse event reporting and risk management		Premises / equipment		Staff safety	
Head nurse and nurse	26	(23)	46	(41)	20	(18)	12	(11)	8	(7)	1	(1)
Nursing assistant	11	(18)	32	(52)	10	(16)	2	(3)	3	(5)	3	(5)
Physician	6	(24)	8	(32)	1	(4)	7	(28)	3	(12)	0	(0)
Other healthcare provider	8	(80)	1	(10)	1	(10)	0	(0)	0	(0)	0	(0)
Administrative	32	(80)	4	(10)	3	(8)	1	(3)	0	(0)	0	(0)
Technical	2	(66)	0	(0)	0	(0)	0	(0)	1	(33)	0	(0)
Head nurse and nurse	26	(23)	46	(41)	20	(18)	12	(11)	8	(7)	1	(1)

*Values were missing for seven comments

## Discussion

Through the analysis of the comments and interviews, we identified several structural failures that could explain the very low scores obtained for certain of the HSOPS dimensions. The lack of resources was identified by all categories of staff members. This could be strongly related to the low dimension scores for “hospital management support” and “staffing.” Moreover, the vast majority of comments reported important problems of communication between units and departments, related to the penultimate dimension score for “hospital handoffs & transitions.” Highlighting concrete failures in the organization and management, the open comments analysis provides a better understanding of the dimension scores computed from the 42 closed questions of the HSOPS. Too rarely done in this type of survey, consideration of the open comments could also help complete the closed questionnaire and target concrete failures in the hospital organization and management.

First, the comments on the survey and the staff members interviewed reported a global lack of resources. Insufficient resources concerned all aspects of their work: staffing, equipment, resources devoted to training, and bed availability. This resulted in an increased level of fatigue, stress, and mental load. Understaffing favored task interruptions, which were considered a significant risk for patients. Insufficient training was said to increase the risk of error but also the level of stress. Bed availability was raised by the interviewees, which degraded patient safety by creating situations in which departments had to handle patients they were not trained or equipped to handle or by making it necessary to resort to corridor beds. The negative effects of lack of training, staffing and equipment issues, as well as the related fatigue, stress, and mental load are widely known and these factors tend to decrease patient safety and the quality of care.[12–15] The deterioration of working conditions (and the subsequent quality of working life) as well as the quality and safety of care, as perceived by the staff members interviewed, encourage medical and paramedical staff to quit their jobs. Several interviewees admitted their desire to resign. High turnover has an impact on cost and skill preservation and creates disorganization. Indeed, departing staff members need to be replaced and new recruits need to be trained so they can work well within the department. Both replacement and training have significant costs.[12,16] The impact of a high turnover rate and staff dissatisfaction over patient satisfaction is not to be neglected and is associated with a decrease in patient safety and quality of care.[17,18] Even though this lack of resources was the major concern for the majority of all occupational groups, this perception was exacerbated for nurses and nursing assistants. This finding was consistent with other studies conducted in Europe and the US reporting understaffing and lack of resources as a major barrier for nurses’ perception of quality of care.[19,20] More generally, qualitative analysis of open comments could help managers identify the most vulnerable groups and could be a useful indicator to track disparity trends and progress over time.

Second, a problem of communication and cooperation between units and departments was identified. This problem resulted in loss of information during transmission or an absence of transmission. It also participated in patient transfer problems. Although several comments argued that patient transfers were guided by defined written procedures, it was obvious that these procedures did not produce solutions to limit the problem occurring during transitions between hospital departments. Therefore, the comments analysis made it possible to focus on concrete problems caused by these procedures, whether related to a lack of dissemination to the wards or to the impossibility of dealing with complex transfer situations. Finally, this tended to underline that other enhancements can be explored.

Third, several open comments reported difficulties related to adverse event reporting, which is of utmost importance because it allows the organization to learn from its errors. Nevertheless, several studies demonstrated that many incidents were not actually reported [21,22]. The majority of reporting systems are based on healthcare professionals’ self-reports, and it is difficult to properly identify the obstacles to self-reporting. Our qualitative data confirmed that lack of feedback and time constraints were the two main barriers. Time constraints could be related to insufficient resources; the lack of feedback was closely related to the organization of risk management. Indeed, the vast majority of French hospitals have a centralized incident-reporting system. Interestingly, this perception is not collected with the closed questions of the HSOPS. The use of open comments could thus help identify specific organizational failures and elaborate tailored solutions to enhance the management of patient safety. Improving feedback after incident reporting does not require major resources and could be accomplished through organizational changes. For example, the staff interviewed raised the possibility of decentralizing risk management in hospital departments by promoting programs directly involving the medical teams in patient safety, such as experience feedback committees and the morbidity and mortality conferences. [23,24]

Besides targeting concrete ways to improve organization of the hospital site, the open comments and interviews provided a number of elements critical of hospital management’s support for patient safety. The very low score for the “hospital handoffs and transitions” dimension is observed in the majority of HSOPS surveys in both Europe and the United States, showing a weakness shared by all organizations.[5,7,25–29] Interestingly, this does not extend to the “hospital management support” dimension, which is nearly always one of the lowest dimension scores in the HSOPS survey conducted in Europe, whereas it is one of the highest dimensions in the United States.[5,7,25–29] Although true contextual differences between the US and Europe might explain inconsistency, the results reported herein have provided food for thought on the priority policy of a French university hospital, which partly explains this difference observed for the “hospital management support” dimension.[30] According to the professionals interviewed, the lack of resources was partly due to management policy, which demands an increase in hospital activity without increasing the available resources, including bed capacity. However, we cannot exclude that the open comments section was used to express complaints that went far beyond the concerns of safety culture. Indeed, the surprising level of anger and resentment of certain comments might suggest that the HSOPS-based survey was used as a sounding board for some of the respondents. This hypothesis raises questions about the reliability of the HSOPS results. Whether the HSOPS score differences observed between the respondents who wrote a comment or did not reflected true patient safety culture differences or information bias remains unclear. Although speculative, these questions show that the qualitative approach adds richness to the classical quantitative approach used for the HSOPS.

This study was conducted in a single hospital, its main limitation. Therefore, not all results can be applied to other sites. However, some of the findings may be applied to sites with a comparable context. A second limitation is the small number of interviews. However, these interviews were conducted in addition to the comments analysis. In that sense, we had rich data and were able to reach data saturation. Finally, we did not explore the different perceptions of the professional categories in depth. Even though we provided a stratified quantitative analysis of the topics in relation to these categories, it would be interesting to focus a qualitative analysis on this question. It cannot be excluded that true differences in education, activity, and experience specific to different professions could strongly influence the respondent’s perception.

## Conclusion

Through a hospital-wide survey, the analysis of open comments provided a better understanding of the results of the HSOPS closed questions. The lack of resources was perceived as the major barrier to improving patient safety and was strongly related to the support provided by hospital management, which was the worst dimension computed by the closed questions. The open comments also identified a number of specific failures related to patient transfers and risk coordination. Mixing qualitative and quantitative approaches in the HSOPS-based surveys could help not only to achieve a better understanding of the HSOPS scores, but also to develop corrective actions to enhance the patient safety culture of healthcare professionals.

## Supporting information

S1 Table Open comments database (in French). (DOCX)

S2 Table Aggregated closed questions database. (DOCX)
