# Supplementary material for: The contribution of open comments to understanding the results from the Hospital Survey on Patient Safety Culture (HSOPS): A qualitative study
Source: PLoS One. 2018 Apr 19;13(4):e0196089. doi: 10.1371/journal.pone.0196089 (PMC5908235; doi:10.1371/journal.pone.0196089)
Supplement: S1 Table — (DOCX) [file pone.0196089.s001.docx]

S1 Table – Open comments database (in French)

| ID | Verbatim | Catégorie | Mots-clés |
| --- | --- | --- | --- |
| 1 | La sécurité des soins nécessite de la rigueur et une vigilance permanente en raison de la lourdeur de la charge de travail et du changement fréquent du personnel. | Ressources humaines et soutien du management | charge de travail / turnover |
| 2 | Questionnaire pas vraiment anonyme ! | Questionnaire | anonyme |
| 3 | Il y a de grosses lacunes dans la prévention d'escarre dans tous les services de soins ; il y a des problèmes dans la mise en place de nouveaux matériels de soins et des consommables qui devraient suivre avec (ex : on a reçu le nouveau dispositif d'aiguilles pour les chambres implantées mais pas les "psts" qui vont avec, idem pour les "piceline" (?) | Organisation | lacunes dans la prévention d'escarres |
| 3 | Il y a de grosses lacunes dans la prévention d'escarre dans tous les services de soins ; il y a des problèmes dans la mise en place de nouveaux matériels de soins et des consommables qui devraient suivre avec (ex : on a reçu le nouveau dispositif d'aiguilles pour les chambres implantées mais pas les "psts" qui vont avec, idem pour les "piceline" (?) | Environnement et matériel | nouveaux matériels / consommables |
| 4 | Le manque de personnel est récurrent dans notre service. Arrêts maladie non remplacés ! Les aides-soignantes ne sont pas payées en heures supplémentaires donc ne veulent pas venir ! Normal ! On demande aux équipes le même travail mais avec un effectif réduit en réanimation vu l'état de santé précaire et grave de nos patients !!! | Ressources humaines et soutien du management | manque de personnel/ remplacements |
| 5 | Participation à la cellule qualité | Questionnaire | précision |
| 6 | Je ne me suis pas sentie concernée à l'égard de ce questionnaire, pas adapté aux secrétaires | Questionnaire | non concernée |
| 7 | Ce questionnaire n'est pas réellement anonyme ! Trop facile de déduire qui l'a rempli | Questionnaire | anonymat |
| 8 | Le CLIN ne passe pas souvent | Evénements indésirables associés aux soins et coordination de la gestion du risque | CLIN |
| 9 | En tant qu'AS nous travaillon souvent seules lors de l'horaire de garde (14h à 21h30). Ceci peut porter préjudice à la sécurité et soins des patients | Organisation | nous travaillons souvent seules |
| 10 | A2 : Variable selon les années si congé maternité ou pas | Questionnaire | précision |
| 11 | Dans la mesure ou chaque agent ferait son travail, le service tournerait beaucoup mieux. Il y a toujours des agents qui comptent sur leurs collègues pour faire leur travail. Il y a un fort manque d'organisation. Quelle est la différence entre un agent responsable et un agent qui n'a aucun respect de ses collègues et de la hiérarchie ? | Organisation | manque d'organisation |
| 12 | En six ans les conditions de travail se sont fortement dégradées. Il est encore plus difficile de travailler correctement. | Ressources humaines et soutien du management | dégradation des conditions de travail |
| 13 | Q A7 : "Jamais" | Questionnaire | précision |
| 14 | Trop souvent la variable est le 'temps' médecin lors de surcroît d'activité. | Ressources humaines et soutien du management | le 'temps' médecin |
| 15 | STRESS-INSECURITE- quand le personnel est déployé dans d'autres services (CA, travaux… (c'est inhumain° | Organisation | redéploiments / stress |
| 16 | Je ne me situe pas au niveau d'un service et d'une unité. Le questionnaire est donc hors de propos en ce qui me concerne. | Questionnaire | non concerné |
| 17 | 2 aides-soignantes pour 18 patients de réanimation… Aide-soignante en sous effectif depuis 2 mois, IDE avec débit crédit à 140h (hors CA) sécurité ? Professionalisme ? Respect des patients ? Merci de nous avoir demandé l'équipe sature, on attend des actions avant nos demandes de mutations. | Ressources humaines et soutien du management | pmanque de personnel |
| 18 | Dégradation continue et inexplicable de la qualité des soins et de la sécurité des patients, malgré quelques actions qu'il faut souligner sur l'hygiène et la transfusion qui sont bien faites. | Evénements indésirables associés aux soins et coordination de la gestion du risque | Dégradation de la qualité et de la sécurité |
| 19 | Notre service est très lourd (que ce soit au B ou C) et avons beaucoup de personnes en soins palliatifs. Notre cadre supérieur considère que ce n'est pas des soins palliatifs ! Il a donc une charge de travail très importante que ce soit pour les aides-soignantes et IDE. Nous manquons de personnels ! Nous IDE effectuons le travail de notre cadre de santé en commandant les médicaments, les pansements, l'oxygène ! Une cadre qui arrive tous les jours à 7h30, repart à 12h pour manger et revient à 14h30 à 17h... Nous n'avons pas l'impression d'être soutenue et écoutée ! | Ressources humaines et soutien du management | charge de travail / manque de personnel |
| 20 | Une bonne communication entre les différents personnels et les services serait la base d'une réduction importante des évènements indésirables | Organisation | bonne communication |
| 21 | Ne concerne pas le personnel administratif | Questionnaire | non adapté |
| 22 | Ce questionnaire n'est pas adapté à notre activité d'équipe mobile transversale, mais j'ai souvent été témoin et à l'écoute des soignants en difficulté face aux risques potentiellement encourus par les patients dont ils avaient la charge (faute de temps, de formation, de matériel, mauvaise organisation, manque personnel...) | Ressources humaines et soutien du management | manque de personnel /formation /temps |
| 22 | Ce questionnaire n'est pas adapté à notre activité d'équipe mobile transversale, mais j'ai souvent été témoin et à l'écoute des soignants en difficulté face aux risques potentiellement encourus par les patients dont ils avaient la charge (faute de temps, de formation, de matériel, mauvaise organisation, manque personnel...) | Organisation | mauvaise organisation |
| 22 | Ce questionnaire n'est pas adapté à notre activité d'équipe mobile transversale, mais j'ai souvent été témoin et à l'écoute des soignants en difficulté face aux risques potentiellement encourus par les patients dont ils avaient la charge (faute de temps, de formation, de matériel, mauvaise organisation, manque personnel...) | Environnement et matériel | manque de matériel |
| 23 | Pas forcément adapté aux différentes catégories professionnelles. Pas de questions sur le matériel à disposition pour travailler dans des conditions de sécurité pour le patient : manutention… | Questionnaire | pas forcément adapté |
| 24 | Je pense que le risque d'erreurs et d'insécurité est lié essentiellement au manque de personnel par manque de temps auprès des patients, ainsi que les plannings surchragés et en 3x7 qui augmentent la fatigue des agents et donc le risque de faute. | Ressources humaines et soutien du management | manque de personnel / temps /fatigue |
| 25 | Le redéploiement est une perte de qualité pour les patients et engendre des risques à tous les niveaux car nous n'avons pas la connaissance suffisante ni des lieux, ni des pathologies pour assurer des soins de qualité. On se sent en danger l'été (lors des changements) | Ressources humaines et soutien du management | redéploiement |
| 26 | Le manque de personnel, plus les nombreux arrêts maladies pas remplacés entrainent de gros disfonctionnement dans la qualité et sécurité des soins, vis-à-vis des patients familles et du personnel soignant. Le manque de personnel met le cadre en difficulté ce qui l'oblige à faire appel à des intérimaires, agents remplaçants et au personnel du service restant. | Ressources humaines et soutien du management | manque de personnel / remplacements / intérimaires |
| 27 | La principale source permanente de tension entre équipe voire de disfonctionnement c'est les effectifs : parfois insuffisant par rapport à la charge de travail, parfois complètement déséquilibrés par le non remplacement des arrêts maladie, 250% sur 2 mois en moins c'est arrivé, c'est scandaleux mais nous sommes dans un service de gériatrie. La 1ère source de problème voire de maltraitance est institutionnelle | Ressources humaines et soutien du management | manque de personnel / remplacements |
| 28 | Questionnaire non adapté à notre structure dont la mission principale est d'aider les équipes soignantes à améliorer leur qualité de soins et de prise en charge de patients porteur de maladie chronique par la mise en place de programmes ETP autorisés par l'ARS. Nous n'accueillons par de patients dans nos bureaux et n'avons pas de contact avec eux | Questionnaire | non adapté |
| 29 | Certaines questions sont difficiles à comprendre : A5 F2 F6 | Questionnaire | difficultés |
| 30 | Etant ASH je n'ai pas répondu aux questions concernant les soins car je n'en pratique pas, et n'ai donc pas d'avis sur ce sujet. | Questionnaire | non adapté |
| 31 | Je ne travaille pas dans un service de soin donc mes réponses neutres corresponent à une question qui n'est pas appropriée | Questionnaire | non adapté |
| 32 | Questionnaire peu adapté à mon poste : celui d'IDE de cs gastro, spécificité du profil de poste : onco digestif ; poste transversal entre plusieurs équipes et plusieurs unités fonctionnelles => manque de communication entre nous tous et poste unique. Je n'aime pas l'anonymat du questionnaire | Organisation | manque de communication |
| 33 | Il est plus que temps d'embaucher du personnel voir de créer des postes si tous sont pourvus !!! | Ressources humaines et soutien du management | manque de personnel |
| 34 | Nous ne sommes pas en unité de soins, ce questionnaire ne nous correspond pas vraiment. Désolé. Cordialement | Questionnaire | non adapté |
| 35 | Le surplus de patient entraîne une baisse de vigilance et donc un risque plus important de survenues d'évènements indésirables. Surplus de patients = augmentation de la charge de travail = baisse de la sécurité dans les soins. | Ressources humaines et soutien du management | charge de travail |
| 36 | Pour les questions 17, 18 et 19 du paragraphe A, liées à la sécurité des soins, je pointe les dangers que représentent les "lits couloirs" = pas de sonnette, pas de prise électrique. | Organisation | lits couloirs |
| 37 | Secteurs très spécialisés, implique difficile de se remplacer et charge de travail parfois supérieure au nombre de personnel. | Ressources humaines et soutien du management | charge de travail / remplacements |
| 38 | Ce questionnaire ne s'adresse pas à ma catégorie professionnelle (aucun soin). | Questionnaire | non adapté |
| 39 | Lorsque l'on fait pas partie d'une petite équipe ce questionnaire n'est absolument pas anonyme ! | Questionnaire | anonymat |
| 40 | Les pertes de temps occasionnées par les procédures, l'absence d'interlocuteur disponible et en charge de lar responsabilité, problème précis pour en suite que la plupart du temps, les évènements préjudiciables ne soient pas portés à la connaissance de l'institution. | Organisation | procédures |
| 40 | Les pertes de temps occasionnées par les procédures, l'absence d'interlocuteur disponible et en charge de lar responsabilité, problème précis pour en suite que la plupart du temps, les évènements préjudiciables ne soient pas portés à la connaissance de l'institution. | Evénements indésirables associés aux soins et coordination de la gestion du risque | Evénements non signalés |
| 41 | Chambres de moins de 5m2, sans fenêtre, pas de wc, de placard. Pas de place pour circuler autour du lit pour faire les soins : pas correct pour accueillir des patients et dangereux si besoin de faire des soins en urgence. De plus les patients sous oxygénothérapie ou peu valides n'ont pas d'intimité quand il y a besoin d'aller sur la chaise pot (impossible de fermer la porte car chambre trop petite). Patient qui remonte du PU en lit couloir, avec VNI 24h/24 sans prise dans le couloir ou besoin d'être aspiré (car ecombré) sans système d'aspiration dans le couloir : comment faire pour la sécurité du patient ? Manque de matérie : impossible de travailler. Réseau informatique qui n'est pas accessible dans le couloir : impossible de faire le tour correctement. | Organisation | lits couloirs |
| 41 | Chambres de moins de 5m2, sans fenêtre, pas de wc, de placard. Pas de place pour circuler autour du lit pour faire les soins : pas correct pour accueillir des patients et dangereux si besoin de faire des soins en urgence. De plus les patients sous oxygénothérapie ou peu valides n'ont pas d'intimité quand il y a besoin d'aller sur la chaise pot (impossible de fermer la porte car chambre trop petite). Patient qui remonte du PU en lit couloir, avec VNI 24h/24 sans prise dans le couloir ou besoin d'être aspiré (car ecombré) sans système d'aspiration dans le couloir : comment faire pour la sécurité du patient ? Manque de matérie : impossible de travailler. Réseau informatique qui n'est pas accessible dans le couloir : impossible de faire le tour correctement. | Environnement et matériel | locaux / informatique |
| 42 | Pas assez de personnel, pas assez de moyens pour faire le travail correctement et surtout pas de prime de risque quand on se fait frapper par des patients car service violent. | Ressources humaines et soutien du management | manque de personnel |
| 42 | Pas assez de personnel, pas assez de moyens pour faire le travail correctement et surtout pas de prime de risque quand on se fait frapper par des patients car service violent. | Sécurité du personnel | violence patient |
| 43 | H9 : RMM | Evénements indésirables associés aux soins et coordination de la gestion du risque | RMM |
| 44 | Manque de personnel pour travailler correctement. Il n'est pas normal de se retrouver à 2 au lieu de 3 parce que les personnes sont en arrêt de travail et non remplacés. Ce qui se répercute forcement sur la prise en charge des patients. Manque de personnel = travail non fait ou fait à moitié. L'efficacité ne riment pas avec rapidité. Il faudrait d'abord mieux gérer le personnel avant de gérer les risques qui du coup sont inévitables. | Ressources humaines et soutien du management | manque de personnel / remplacements |
| 45 | Nous sommes une équipe soudée et nous mettons tout en œuvre pour le bien être de nos résidents. Je me sens très soutenue par toute l'équipe que ce soit ASH, AS, IDE, cadre et médecin. Peut-être un manque de moyen certains jours, mais l'ambiance conviviale fait de La Bâtie une maison de retraite très agréable à vivre. | Organisation | conviviale / soutien |
| 46 | Très bonne relation de travail au sein de toute l'équipe, tous postes confondus. Maison de retraite agréable. | Organisation | bonnes relations de travail |
| 47 | Beaucoup d'insécurité surtout la nuit | Sécurité du personnel | Insécurité |
| 48 | La sécurité des patients est parfois engagée pour certains passages d'examen, notamment les scanners : retour des patients sans voie veineuse, équipement endommagé ou mal remis en place, relation avec les patients très difficile avec le personnel de ces lieux d'examens. Evidemment ce n'est pas un fait isolé mais pour autant on ne peut mettre tout le personnel dans le même panier. | Organisation | Transfert en imagerie |
| 49 | L'exercice de ma profession n'espose pas le patient à un risque immédiat direct à l'exclusion d'un contexte d'allergie alimentaire. Je n'évalue pas aussi justement qu'un autre soignant l'exposition à un risque au sein du service dans lequel j'interviens | Questionnaire | non adapté |
| 50 | Questionnaire avec les informations générales devient non anonyme | Questionnaire | anonymat |
| 51 | A7 : 'ce n'est pas le cas !' | Questionnaire | précision |
| 52 | A quoi cela va-t-il servir ? Plus de personnels ? | Questionnaire | utilité ? |
| 52 | A quoi cela va-t-il servir ? Plus de personnels ? | Ressources humaines et soutien du management | manque de personnel |
| 53 | Pour moi la réponse neutre = ça dépend | Questionnaire | précision |
| 54 | Ca ne fait pas assez longtemps que je suis à ce poste pour répondre aux questions ! Temps 50% depuis mars, arrêt 2 mois. | Questionnaire | non adapté |
| 55 | A quoi ça sert ? | Questionnaire | utilité ? |
| 56 | La sécurité des soins et des patient découle souvent d'un travail qui peut s'effectuer dans des conditions correctes… ce qui ne semble pas le cas actuellement… Trop d'effectifs nouveaux, des demandes très exigeantes en terme de quantité et de qualité de travail, et une pénurie chronique de personnel... Quant au signalement d'erreur il est fait mais c'est encore insuffisant, beaucoup de choses passent à l'as... | Ressources humaines et soutien du management | manque de personnel /turnover |
| 56 | La sécurité des soins et des patient découle souvent d'un travail qui peut s'effectuer dans des conditions correctes… ce qui ne semble pas le cas actuellement… Trop d'effectifs nouveaux, des demandes très exigeantes en terme de quantité et de qualité de travail, et une pénurie chronique de personnel... Quant au signalement d'erreur il est fait mais c'est encore insuffisant, beaucoup de choses passent à l'as... | Evénements indésirables associés aux soins et coordination de la gestion du risque | signalement insuffisant |
| 57 | dans ce service nombreux outils de gestion du risque: CREX, RMM; politique hospitalière de gestion du risque très mal lisible | Evénements indésirables associés aux soins et coordination de la gestion du risque | présence d'outils sécurité |
| 58 | Le sous effectif permanent du pesonnel contribue à la déterioration et baisse de la vigilence et des capacités lors des soins. | Ressources humaines et soutien du management | manque de personnel |
| 59 | Le problème aujourd'hui est la charge de travail dûe au manque de personnel. Je ne suis pas suûre que les équipes tiendront le choc longtemps. Personnellement je viens d'arriver il y a plusieurs mois et je ressens déjà la fatigue dûe à la charge de travail. | Ressources humaines et soutien du management | manque de personnel / fatigue |
| 60 | Manque important de personnel, donc moins de motivation pour exercer notre métier. | Ressources humaines et soutien du management | manque de personnel / motivation |
| 61 | Une augmentation de personnel de chaque catégorie serait la bienvenue. Quelle utopie !!! Il y aurait moins de tension lors des crises. Les remplacements de congés d'été devraient être pris plus en considération. On pourrait partir 3 semaines consécutives donc se reposer donc récupérer d'ou une meilleure prise en compte au niveau sécurité. | Ressources humaines et soutien du management | manque de personnel / tensions / remplacements |
| 62 | L'unité ne fait que des consultations pour les patients ambulatoires ou hospitalisés. Pas de lits d'hospitalisation. | Questionnaire | non adapté |
| 63 | Quelle finalité ? Aura-t'on un retour ? | Questionnaire | utilité ? |
| 64 | Etant secrétaire médicale dans le service, j'ai été en difficulté pour répondre à cetette enquête pour la sécurité des soins. | Questionnaire | non adapté |
| 65 | A quoi sert ce type d'enquête ? | Questionnaire | utilité ? |
| 66 | En tant qu'animatrice je ne suis pas directement impliquée dans le soin et il m'est difficile de répondre correctement à ce questionnaire. | Questionnaire | non adapté |
| 67 | Plus axé au personnel soignant qu'aux administratifs d'où beaucoup de réponse neutres | Questionnaire | non adapté |
| 68 | Nous sommes dans un processus de rentabilité, de productivité, c'est bien dommage. Certes l'hôpital est une entreprise mais on gère les humains et là est toute la différence. | Ressources humaines et soutien du management | politique /rentabilité |
| 69 | Questionnaire non adapté au métier | Questionnaire | non adapté |
| 70 | En tant que secrétaire je ne peux pas répondre à ces différentes questions inhérentes surtout à l'équipe soignante. | Questionnaire | non adapté |
| 71 | Dans notre service, chaque jour nous avons du personnel intérimaire différent, ce qui provoque une surcharge de travail, une mauvaise prise en charge des patients, des pertes d'informations, une fatigue physique et psychologique avec des risques de blessures corporelles importantes (patient violent). | Ressources humaines et soutien du management | charge de travail / interimaires /fatigue |
| 71 | Dans notre service, chaque jour nous avons du personnel intérimaire différent, ce qui provoque une surcharge de travail, une mauvaise prise en charge des patients, des pertes d'informations, une fatigue physique et psychologique avec des risques de blessures corporelles importantes (patient violent). | Sécurité du personnel | patients violents |
| 72 | Des parapluies sont ouvers pour la direction de l'établissement sans vraiment prendre en compte les problèmes de fonds : se protége au détriment des personnels, l'erreur la faute est rejetée sur l'agent. Maltraitance des agents, mals considérés par la direction. | Ressources humaines et soutien du management | Maltraitance des agents / considération |
| 72 | Des parapluies sont ouvers pour la direction de l'établissement sans vraiment prendre en compte les problèmes de fonds : se protége au détriment des personnels, l'erreur la faute est rejetée sur l'agent. Maltraitance des agents, mals considérés par la direction. | Evénements indésirables associés aux soins et coordination de la gestion du risque | erreur = faute |
| 73 | Pour le 1/4 h qualité, dégager 1 IDE + 1 AS du service + la cadre de santé - Comment participer au CREX et au RMM en tant qu'IDE ? - La recherche infirmière améliore la qualité, la sécurité et l'efficience des soins. Comment former plus d'IDE en master sciences cliniques infirmières ? | Ressources humaines et soutien du management | temps |
| 73 | Pour le 1/4 h qualité, dégager 1 IDE + 1 AS du service + la cadre de santé - Comment participer au CREX et au RMM en tant qu'IDE ? - La recherche infirmière améliore la qualité, la sécurité et l'efficience des soins. Comment former plus d'IDE en master sciences cliniques infirmières ? | Evénements indésirables associés aux soins et coordination de la gestion du risque | formation |
| 74 | Ne suis pas concernée pas ce questionnaire (directement) | Questionnaire | non adapté |
| 75 | Le questionnaire de l'agent qui a rempli ce document me laisse entendre que celui-ci n'est pas tout à fait anonyme. Le manque de personnel agit sur la sécurité des soins et reste le problème majeur dans l'ensemble de l'établissement. | Ressources humaines et soutien du management | manque de personnel |
| 76 | Rapidité ++ Cadence peut nuire à la sécurité du patient et des soignants. Pour les soignants récurrent au niveau relationnel ++ Vigilance réduite. Service qui à ce jour en constante évolution de ses besoins réels et en constat des efforts indésirables, capacité, besoins humains. | Ressources humaines et soutien du management | manque de personnel /cadences |
| 77 | H9 = CRUQ. Travail de coordination à faire entre les services (lors de mutation de patient) et avec la pharmacie. Décalage entre les axes de travail du CHU (les différents comités de lutte # risque) et la réalité concrète au quotidien. | Organisation | Mutation de patient |
| 77 | H9 = CRUQ. Travail de coordination à faire entre les services (lors de mutation de patient) et avec la pharmacie. Décalage entre les axes de travail du CHU (les différents comités de lutte # risque) et la réalité concrète au quotidien. | Evénements indésirables associés aux soins et coordination de la gestion du risque | Coordination |
| 78 | La sécurité passe avant tout pour un nombre de personnel suffisant et une meilleure communication entre les différents corps de métier ! Il faut aussi avoir un matériel adapté : sac DASRI qui se percent systématiquement depuis le changement de marché ! | Ressources humaines et soutien du management | manque de personnel |
| 78 | La sécurité passe avant tout pour un nombre de personnel suffisant et une meilleure communication entre les différents corps de métier ! Il faut aussi avoir un matériel adapté : sac DASRI qui se percent systématiquement depuis le changement de marché ! | Organisation | communication |
| 78 | La sécurité passe avant tout pour un nombre de personnel suffisant et une meilleure communication entre les différents corps de métier ! Il faut aussi avoir un matériel adapté : sac DASRI qui se percent systématiquement depuis le changement de marché ! | Environnement et matériel | matériel non adapté |
| 79 | Il faut multiplier les efforts pour chacun de nous et participer ++ Plus de réunions à l'avenir. | Organisation | participation |
| 80 | Manque de personnel. | Ressources humaines et soutien du management | Manque de personnel. |
| 81 | Service gigantesque, mission de cadre de proximité impossible à effectuer : 60 agents / 64 résidants (quand collègue malade ou en congés, compter le double). Nombreux dysfonctionnements (très) anciens, récurrents avec pires de 'pire'. Heureusement il y a de (très) bons soignants qui font en sorte que les résidants soient bien tenus. Sentiment d'insécurité pour moi, dans une fonction immense. Perception // d'insécurité pour les résidants. C'est terrible. | Ressources humaines et soutien du management | cadre débordé |
| 82 | Drôle de questionnaire | Questionnaire | utilité ? |
| 83 | Manque de personnel pour un service lourd (1 IDE pour 40 patients, patients de plus en plus lourds). | Ressources humaines et soutien du management | manque de personnel |
| 84 | Commentaires A2 ; ça dépend des jours; A17:Je ne me suis jamais posé la question et ne sait pas ce qu'en pense le personnel en général;E: "Bon!!!";F4:ça dépend des services;F5:effet aggravé en cette période semi informatisée!!!;F8:les bonnes idées et les formations c'est chouette mais la ? du sous effectif tend à prouver que la sécurité n'est une priorité théorique | Ressources humaines et soutien du management | manque de personnel |
| 84 | Commentaires A2 ; ça dépend des jours; A17:Je ne me suis jamais posé la question et ne sait pas ce qu'en pense le personnel en général;E: "Bon!!!";F4:ça dépend des services;F5:effet aggravé en cette période semi informatisée!!!;F8:les bonnes idées et les formations c'est chouette mais la ? du sous effectif tend à prouver que la sécurité n'est une priorité théorique | Environnement et matériel | informatique |
| 85 | en raison de ma fonction de secrétaire, je ne peux répondre à la plupart des questions de cette enquête, destinée plutôt aux services de soins médicaux. | Questionnaire | non adapté |
| 86 | Ce questionnaire ne rentre pas vraiment dans le cadre du travail d'une secrétaire médicale. | Questionnaire | non adapté |
| 87 | en tant qu'ASH, nous ne savons strictement rien sur les patients, c'est dommage | Organisation | communication |
| 88 | la culture de la sécurité est bien développée parmi les soignants mais elle est sciemment dégradée et battue en brèche par le sous-effectif le plus souvent absurdement imposé | Ressources humaines et soutien du management | manque de personnel |
| 89 | Etant ASH et travaillant à 50% je n'ai pas trop de contact avec l'équipe soignante et les patients. De plus cela fait moins d'un an que je suis dans le service. Pas évident de répondre aux questions qui touchent beaucoup à la sécurité des soins. | Questionnaire | ancienneté |
| 90 | Le questionnaire n'est pas adapté à notre corps de métier | Questionnaire | non adapté |
| 91 | G : réponse A car bénéfice nul, H9 réponse B pas le temps sur le temps de travail théorique10/10/2013 | Questionnaire | précision |
| 92 | Il n'est pas possible de ne pas se sentir concerné lorsqu'une erreur est détectée, même si le problème est institutionnel. Où se situe la limite entre sécurité des sins et rapidité dans notre travail ? Par manque de temps, la PEC des patients est souvent superficielle et donc insécurisante (recherche antécédents différentes pathologies,...) | Ressources humaines et soutien du management | manque de temps |
| 93 | Avoir une meilleure cohésion au sein de l'équipe | Organisation | meilleure cohésion |
| 94 | Beaucoup de questions sur les soins, difficile de répondre pour une secrétaire. | Questionnaire | non adapté |
| 95 | Il faudrait être plus de personnel surtout l'après midi en UHR principalement car les risques liés aux aléas comportementaux sont très forts | Ressources humaines et soutien du management | manque de personnel |
| 95 | Il faudrait être plus de personnel surtout l'après midi en UHR principalement car les risques liés aux aléas comportementaux sont très forts | Sécurité du personnel | violences patient |
| 96 | Rétablir la salubrité des escaliers : depuis l'interdiction du tabagisme cet endroit est devenu un endroit de fumeur et devenu insalubre : bouteilles d'eau marron qui y stagne ensemble avec mégots, gants de xx, masques, mégots par terre + tasses de café plus ou moins vides parfois renversées, mégots dans l'aération... --> risque d'infection nosocomilale. Aucune privacy aux urgences, par manque de place manque de personnel --> insalubrité aux urgences : irrespect des malades du coup... | Ressources humaines et soutien du management | manque de personnel |
| 96 | Rétablir la salubrité des escaliers : depuis l'interdiction du tabagisme cet endroit est devenu un endroit de fumeur et devenu insalubre : bouteilles d'eau marron qui y stagne ensemble avec mégots, gants de xx, masques, mégots par terre + tasses de café plus ou moins vides parfois renversées, mégots dans l'aération... --> risque d'infection nosocomilale. Aucune privacy aux urgences, par manque de place manque de personnel --> insalubrité aux urgences : irrespect des malades du coup... | Environnement et matériel | locaux / insalubrité |
| 97 | Questionnaire très accès aux soignants, vive la sécurité des soins ! D'où le nombre de réponses neutres. Je pense qu'il serait intéressant d'en avoir un pour les administratifs : relation entre collègues, aide mutuelle, charge de travail entraînant du stress de la colère... Erreurs commises du fait d'un travail qui doit être fait vite peu importe comment... | Ressources humaines et soutien du management | charge de travail stress |
| 98 | Service qui tient compte et qui applique les règles de sécurité et toutes autres mesures, protocoles etc | Organisation | applique les règles de sécurité |
| 99 | Certaines questions laissent entendre différentes interprétations, donc très difficile de répondre clairement. | Questionnaire | difficultés |
| 100 | Manque de personnel = manque de sécurité | Ressources humaines et soutien du management | manque de personnel |
| 101 | Par moment question pas très claire. | Questionnaire | difficultés |
| 102 | E : 'ne sais pas' | Questionnaire | précision |
| 103 | Comment voulez-vous parler de sécurité quand votre cadre vous rappelle constament sur vos repos, que l'on a l'impossibilité de poser plus de 15 jours de vacances sachant que nous sommes dans un service dur physiquement et psychologiquement. | Ressources humaines et soutien du management | manque de personnel / contraintes |
| 104 | Les départs à la retraite, les congés maternité, les arrêts maladies (fréquents) ne sont pas remplacés, ? la charge de travail met le personnel soignant en difficulté de donner le meilleur de lui même | Ressources humaines et soutien du management | charge de travail / remplacements |
| 105 | En demandant la fonction et le nom du service le questionnaire n'est plus vraiment anonyme, car une seule personne exerce vraiment ma fonction dans ce service. | Questionnaire | anonymat |
| 106 | Nous avons mis en place un CREX en 2008 après un audit de la MCHA que j'avais demandé. | Evénements indésirables associés aux soins et coordination de la gestion du risque | présence d'un CREX |
| 107 | Certaines questions sont incompréhensives ! Veuillez m'excuser de ne pas les répondre ! Merci de votre compréhension ! | Questionnaire | difficultés |
| 108 | L'unité d'acceuil et de soins pour sourds n'est pas un service d'urgence ni d'hospitalisation , c'est pourquoi certaines questions ne me semblent pas concernées. | Questionnaire | non adapté |
| 109 | Ce questionnaire est une très bonne initiative !! En espérant qu'il y ait un retour… | Questionnaire | bonne initiative |
| 110 | Nous sommes déplacées dans d'autres services sans formation et cela est une insécurité pour le patient. Personne ne tient compte du retour négatif et du stress que cela implique, les cadres sup' gère le personnel sans aucun intérêt pour les soins | Ressources humaines et soutien du management | redépoiement / stress / formation |
| 111 | Nous n'avons pas suffisamment de personnel le week-end du matin pour un service lourd. | Ressources humaines et soutien du management | manque de personnel |
| 112 | Je trouve que ce questionnaire est surtout fait pour les infirmiers et infirmières, aide soignant, mais non pour les agents service hospitalier | Questionnaire | non adapté |
| 113 | Gros problème de gestion des patients, surtout quand ils proviennent des urgences. | Organisation | urgences |
| 114 | Questionnaire très orienté vers le personnel paramédical ce qui le rend difficile à remplir pour d'autres professionnels, notamment administratif, encadrement, etc. Les tournures de phrases sont également surprenantes ! | Questionnaire | non adapté |
| 115 | Faisant partie du personnel administratif je ne me sens pas compétente pour répondre au questionnaire. | Questionnaire | non adapté |
| 116 | Attention au transfert des cartes de groupe | Organisation | transfert |
| 117 | QA1 : non faisable ; problèmes de sécurité des soins induits par le domicile ; QH9 : RMM | Questionnaire | précision |
| 118 | L'hôpital se dégrade à tous points de vue, dommage !!! Le personnel n'a pas envie de s'investir dans leur travail, pas de reconnaissance etc,,, et les patients ont de plus en plus peur… | Ressources humaines et soutien du management | reconnaissance |
| 119 | La sécurité des soins va de pair avec la charge de travail. Si celle-ci est trop importante elle va au détriment de la sécurité et de la qualité des soins. Sécurité et rendement ne vont pas ensemble. | Ressources humaines et soutien du management | charge de travail |
| 120 | 3/ Oui il y a une entraide mais encore beaucoup de jeunes diplômées travaillent dans l'affectivité et se déplacent uniquement si ce sont les copines. 5/ Nombreuses absences qui nécessitent des appels récurrents aux IDE et A en repos, heures supplémentaires payées ou non fonction de ??? 13/ Il y a la lecture de la non punition à developper : difficulté d'être confronté à une erreur, difficulté à la déclarer, mentalités à changer. Culture du risque à étendre. Les agents n'ont pas la culture de la santé publique... très égocentrique. Voir les recrutements veulent des services en 12h pour venir le moins souvent et peu d'entre eux participent à des groupes de travail. | Ressources humaines et soutien du management | absenteîsme : heures supplémentaires |
| 120 | 3/ Oui il y a une entraide mais encore beaucoup de jeunes diplômées travaillent dans l'affectivité et se déplacent uniquement si ce sont les copines. 5/ Nombreuses absences qui nécessitent des appels récurrents aux IDE et A en repos, heures supplémentaires payées ou non fonction de ??? 13/ Il y a la lecture de la non punition à developper : difficulté d'être confronté à une erreur, difficulté à la déclarer, mentalités à changer. Culture du risque à étendre. Les agents n'ont pas la culture de la santé publique... très égocentrique. Voir les recrutements veulent des services en 12h pour venir le moins souvent et peu d'entre eux participent à des groupes de travail. | Organisation | solidarité / participation |
| 120 | 3/ Oui il y a une entraide mais encore beaucoup de jeunes diplômées travaillent dans l'affectivité et se déplacent uniquement si ce sont les copines. 5/ Nombreuses absences qui nécessitent des appels récurrents aux IDE et A en repos, heures supplémentaires payées ou non fonction de ??? 13/ Il y a la lecture de la non punition à developper : difficulté d'être confronté à une erreur, difficulté à la déclarer, mentalités à changer. Culture du risque à étendre. Les agents n'ont pas la culture de la santé publique... très égocentrique. Voir les recrutements veulent des services en 12h pour venir le moins souvent et peu d'entre eux participent à des groupes de travail. | Evénements indésirables associés aux soins et coordination de la gestion du risque | culture du blame / signalements |
| 121 | H : "Anonyme = pas de grade, pas de sexe, pas d'âge, etc… etc…" | Questionnaire | anonymat |
| 122 | L'hôpital tire de plus en plus sur la corde (concernant le personnel). H9 : A formations. | Ressources humaines et soutien du management | contraintes |
| 123 | Les évènements indésirables sont "librement" signalés mais non pris en compte | Evénements indésirables associés aux soins et coordination de la gestion du risque | signalements sans effets |
| 124 | Très mécontant du départ de notre cadre | Ressources humaines et soutien du management | départ du cadre |
| 125 | Sécurité des soins + CREX + procédure qualité très bien développée et mise ne œuvre en radiothérapie avec priorité de cette spécialité depuis plusieurs années au niveau national. | Evénements indésirables associés aux soins et coordination de la gestion du risque | très dévellopée |
| 126 | Je fais peu de fiches de signalement car c'est une chose de plus à faire sur nos journées très remplies. En plus cela ne changera rien. Je travaille dans une équipe motivée, qui recherche le meilleur pour le patient, malgré l'énorme surcharge de travail. Je déplore, malgré le grand dévouement de cette équipe, une prise en charge non globale du patient à cause de la surcharge de travail. Il y a donc une baisse de qualité et une baisse de sécurité des soins mais on cherchera toujours à faire 'sécurité 100%'. Nous courons beaucoup au détriment du relationnel, du temps avec le patient, de la qualité... Il faut de la rentabilité, il faut faire toujours plus sans plus de moyens. J'ai perdu l'âme de mon métier. | Ressources humaines et soutien du management | charge de travail /temps |
| 126 | Je fais peu de fiches de signalement car c'est une chose de plus à faire sur nos journées très remplies. En plus cela ne changera rien. Je travaille dans une équipe motivée, qui recherche le meilleur pour le patient, malgré l'énorme surcharge de travail. Je déplore, malgré le grand dévouement de cette équipe, une prise en charge non globale du patient à cause de la surcharge de travail. Il y a donc une baisse de qualité et une baisse de sécurité des soins mais on cherchera toujours à faire 'sécurité 100%'. Nous courons beaucoup au détriment du relationnel, du temps avec le patient, de la qualité... Il faut de la rentabilité, il faut faire toujours plus sans plus de moyens. J'ai perdu l'âme de mon métier. | Evénements indésirables associés aux soins et coordination de la gestion du risque | peu de signalements |
| 127 | Personnel en nombre insuffisant par rapport à la charge de travail, niveau de dépendance et le besoin de la personne agée pour une PEC satisfaisante | Ressources humaines et soutien du management | manque de personnel |
| 128 | Anonymat questionnaire ? | Questionnaire | anonymat |
| 129 | Questionnaire pas bien applicable pour moi en tant que psychologue… | Questionnaire | non adapté |
| 130 | Je n'ai pas compris les questions A5 F2 F6 | Questionnaire | difficultés |
| 131 | Questionnaire inadapté pour les secrétaires médicales | Questionnaire | non adapté |
| 132 | Pas d'organisation et les transmissions où très peu. On nous laisse plus le emps de faire notre travail correctement, il faut aller toujours très vite et plus de rendement avec le nombre de personnes ou seul c'est la même chose. Le patient doit être prioritaire pour les soins et sa sécurité et son confort, mais beaucoup de personnel médical ou paramédical l'oublit. En tant que ASH on nous demande plus notre avis, on nous met au fait accompli pour n'importe quelles tâches et changement de planning. Bonne entente entre ASH, des patients nous font des compliments sur notre travail | Ressources humaines et soutien du management | rendement |
| 132 | Pas d'organisation et les transmissions où très peu. On nous laisse plus le emps de faire notre travail correctement, il faut aller toujours très vite et plus de rendement avec le nombre de personnes ou seul c'est la même chose. Le patient doit être prioritaire pour les soins et sa sécurité et son confort, mais beaucoup de personnel médical ou paramédical l'oublit. En tant que ASH on nous demande plus notre avis, on nous met au fait accompli pour n'importe quelles tâches et changement de planning. Bonne entente entre ASH, des patients nous font des compliments sur notre travail | Organisation | transmissions |
| 133 | Manque de personnels ! Manque de reconnaissance, service lourd psychologiquement et physiquement. | Ressources humaines et soutien du management | manque de personnel / reconnaissance |
| 134 | Ce questionnaire ne rentre pas vraiment dans le cadre du travail d'une secrétaire médicale. | Questionnaire | non adapté |
| 135 | Secrétaire je ne peux répondre facilement à ces questions. Actuellement le service connait de gros problèmes de charge de travail et un gros manque d'effectif dans toutes les catégories de personnels. | Ressources humaines et soutien du management | manque de personnel |
| 136 | 1/ Plusieurs items ambigus.2/ Il parait ridicule de mesurer les situations à risque ou les "évènements indésirables" par le seul compte des fiches : l'absence de matériel de remplacement pour les ?, le ? De problème informatique/délai d'intervention qui augmentait la charge de travail considérablement ne sont que 2 exemples reportant la cause d'augmentation du risque sanitaire. 3/ ? par des fiches les dysfonctionnements est chronophage, ils n'expliquent probablement que la partie émergée de l'iceberg | Environnement et matériel | absence de matériel /informatique |
| 136 | 1/ Plusieurs items ambigus.2/ Il parait ridicule de mesurer les situations à risque ou les "évènements indésirables" par le seul compte des fiches : l'absence de matériel de remplacement pour les ?, le ? De problème informatique/délai d'intervention qui augmentait la charge de travail considérablement ne sont que 2 exemples reportant la cause d'augmentation du risque sanitaire. 3/ ? par des fiches les dysfonctionnements est chronophage, ils n'expliquent probablement que la partie émergée de l'iceberg | Evénements indésirables associés aux soins et coordination de la gestion du risque | Signalements prennent du temps /non exhaustifs |
| 137 | Chambre sans fenêtre ni wc dans 4 chambres au 4A…Pas de ??? Noir dans les chambres du 3è secteur. Lit couleur avec machine ventilation sans prise, sans effectif de personnel… | Organisation | Lit couloir avec machine ventilation sans prise /locaux |
| 137 | Chambre sans fenêtre ni wc dans 4 chambres au 4A…Pas de ??? Noir dans les chambres du 3è secteur. Lit couleur avec machine ventilation sans prise, sans effectif de personnel… | Environnement et matériel | Chambre sans fenêtre |
| 138 | G : répnse A, Fiche de signalement effets indésirables pour les patients inclus dans les protocoles de recherche clinique, uniquement à la pharmacovigilance. | Evénements indésirables associés aux soins et coordination de la gestion du risque | Pas de signalement hormis protocole de recherche |
| 139 | Si l'établissement souhaite améliorer la sécurité du patient à cause des pertes d'informations lors des transmissions, il faudrait que ce temps ne soit pas bénévole et donc moins succinct | Organisation | transmissions |
| 140 | Le manque d'effectif chronique du service nuit gravement à la sécurité du patient : petits soins de confort négligés, équipe surmenée, impossibilité de faire nos soins dans des conditions adéquates. J'ai honte de travailler dans de pareilles conditions, et n'aimerais pas me retrouver dans un de nos lits ! Merci de nous entendre et de faire changer les choses. | Ressources humaines et soutien du management | manque de personnel / Surmenage |
| 141 | Il n'y a pas beaucoup de réponse de ma part étant donné que je ne me retrouve pas dans ces questions n'étatnt pas personnle soignant. Ce questionnaire est une bonne initiative en espérant qu'il aboutisse à certains problèmes récurrents dans cet établissement. il manque des questions concernant le management des équipes et l'écoute. | Questionnaire | bonne initiative |
| 142 | Pas de retour sur les fiches UMAGRIS effectuées dans le service (si problème pris en compte ou non) | Evénements indésirables associés aux soins et coordination de la gestion du risque | Pas de retour sur les signalements |
| 143 | Enquête non adaptée aux laboratoires | Questionnaire | Enquête non adaptée aux laboratoires |
| 144 | Dans le signalement des erreurs on pourrait faire preuve de plus de tact, de respect et dicrétion les uns envers les autres et trancher en communication non violente. | Evénements indésirables associés aux soins et coordination de la gestion du risque | diplomatie du signalement |
| 145 | Qestionnaire trop long, items trop généraux pour les différentes catégoreis professionnelles répondant : manque de sensibilité possible. Pour les fiches UMAGRIS, le service en fait de moins en moins car pas de retour. | Evénements indésirables associés aux soins et coordination de la gestion du risque | retours des signalements |
| 146 | De plus en plus de patients, ils attendent plusieurs heures avant d'être pris en charge. Personnel épuisé, travail à la chaîne. Risque d'erreurs augmenté. | Ressources humaines et soutien du management | Charge de travail / épuisement /attentes |
| 147 | Difficile de se remplacer mutuellement car secteurs très spécifiques et multiples. | Organisation | remplacement |
| 148 | Service très difficile, trop de personnes extérieures au service, aucun suivi des patients, surtout l'après-midi car moins de personnel | Ressources humaines et soutien du management | manque de personnel / personnel extérieur |
| 149 | Manque de personnel pour une charge de travail importante. | Ressources humaines et soutien du management | Manque de personnel |
| 150 | Ce sondage ne s'adresse pas vraiment aux secrétaires… Dommage | Questionnaire | non adapté |
| 151 | Travaille dans un secteur de consultations ; Q pas du tout en relation avec mon poste de travail, cordialement | Questionnaire | non adapté |
| 152 | Mettre e n place une musique de fond dans les couloirs pour détendre les patients. Moins de surcharge de travail. Mise en place d'un distributeur de tickets d'attente pour les patients. | Ressources humaines et soutien du management | charge de travail |
| 152 | Mettre e n place une musique de fond dans les couloirs pour détendre les patients. Moins de surcharge de travail. Mise en place d'un distributeur de tickets d'attente pour les patients. | Environnement et matériel | Salle d’attente |
| 153 | H9 : A formations. | Questionnaire | précision |
| 154 | Le premier facteur causal d'accident est la quantité de travail trop important des infirmières source d'une grande partie des problèmes. | Ressources humaines et soutien du management | charge de travail |
| 155 | Dans notre service nous travaillons vraiment en équipe mais il y a malheureusement un manque de personnel récurrent qui forcement génère du stress et des erreurs. La prise en charge du patient en fin de vie avec des pathologies très lourdes mériterait une équipe avec un effectif plus important. Etre rappelée sur ses repos devient vite très usant. | Ressources humaines et soutien du management | manque de personnel / Stress /contraintes |
| 156 | E : réponse D cause manque de personnel | Ressources humaines et soutien du management | manque de personnel |
| 157 | Trop de corrections peuvent générer d'autres erreurs. Nos sécurités sont très souvent informatiques mais l'amélioration des logiciels ne dépend pas de nous. Les concepteurs ont d'autres chats à fouetter et l'argent reste le nerf de la guerre. | Environnement et matériel | Informatique |
| 158 | Je suis IDE d'éducation thérapeutique, j'interviens ponctuellement dans le service. Je ne peux donc répondre qu'à un minimum de questions désolée ! | Questionnaire | non adapté |
| 159 | H9 : CLIN | Evénements indésirables associés aux soins et coordination de la gestion du risque | CLIN |
| 160 | Petites chambres sans fenêtre, ni toilettes et ni douche. Les patients sont obligés d'utiliser le même endroit pour se laver ou pour aller aux toilettes | Environnement et matériel | locaux |
| 161 | C'est bien de s'inquiéter et de réfléchir ensemble | Questionnaire | bien |
| 162 | En qualité de secrétaire, je ne suis pas informée directement des problèmes survenus dans le service ou l'unité dont je gère le secrétariat. C'est par hasard ou le ouie dire que je l'apprends | Questionnaire | non adapté |
| 163 | Je n'ai jamais entendu parler du CREX ! La culture sécurité des soins doit être améliorée, elle existe, chaque professionnel y participe mais elle devrait être plus concrète (informations aux différents soignants, analyse de pratique, cadre impliqué dans la communication sur la sécurité des soins). Selon moi, la réfléxion/communication entre plusieurs professionnels de santé (ou autres) est fondamentale pour la sécurité des soins, (chacun a sa pierre à apporter), | Evénements indésirables associés aux soins et coordination de la gestion du risque | Implication des personnels dans la gestion de la sécurité des patients |
| 164 | B4 réponse 4 = Quand nous lui faisons part de la charge de travail trop importante et du grand risque d'erreur qui peut arriver. F1 : la charge de travail est trop importante pour l'effectif soignant. F2 : les entrées des urgences arrivent souvent très longtemps après l'heure prévue, le pool arrive aussi très en retard ou pas du tout ! | Ressources humaines et soutien du management | charge de travail, écoute du cadre |
| 164 | B4 réponse 4 = Quand nous lui faisons part de la charge de travail trop importante et du grand risque d'erreur qui peut arriver. F1 : la charge de travail est trop importante pour l'effectif soignant. F2 : les entrées des urgences arrivent souvent très longtemps après l'heure prévue, le pool arrive aussi très en retard ou pas du tout ! | Organisation | horaires d'entrée des urgences |
| 165 | Difficile de répondre pour une secrétaire concerne principalement les soignants. | Questionnaire | non adapté |
| 166 | Rien n'est fait pour nous faciliter les tâches, informatique dysfonctionne pas de place de parking, pas de réunion | Organisation | réunions |
| 166 | Rien n'est fait pour nous faciliter les tâches, informatique dysfonctionne pas de place de parking, pas de réunion | Environnement et matériel | informatique |
| 167 | Le plus grand risque pour le patient est à mon avis généré par le manque de personnel, qui fait défaut dans la plupart des services. Et le matériel est de plus en plus fragile et mal adapté : exemple récent dans le service une tubulure qui se casse spontanément avant fort heureusement sa mise en place sur le PAC d'un patient. | Ressources humaines et soutien du management | manque de personnel |
| 167 | Le plus grand risque pour le patient est à mon avis généré par le manque de personnel, qui fait défaut dans la plupart des services. Et le matériel est de plus en plus fragile et mal adapté : exemple récent dans le service une tubulure qui se casse spontanément avant fort heureusement sa mise en place sur le PAC d'un patient. | Environnement et matériel | matériel inadapté |
| 168 | Peu concernée par la plupart des questions | Questionnaire | non adapté |
| 169 | Quand nous sommes déployés dans d'autres services, nous subissons du stress et les patients le subissent (problèmes d'insécurité +++° | Organisation | redéploiements /stress |
| 170 | La charge de travail est de plus en plus lourde et avec moins de matériel. On ressent le manque de personnel donc l'équipe se fatigue plus vite. On est pas assez à l'écoute | Ressources humaines et soutien du management | charge de travail |
| 170 | La charge de travail est de plus en plus lourde et avec moins de matériel. On ressent le manque de personnel donc l'équipe se fatigue plus vite. On est pas assez à l'écoute | Environnement et matériel | moins de matériel |
| 171 | Les erreurs seraient peut-être limitées si la direction de l'établissement s'investissait plus sur le nombre de pesonnel présents. On limite le personnel, moins de temps pour le patient. Des erreurs car il faut uand même faire les soins… spirale. | Ressources humaines et soutien du management | contrintes sur le personnel |
| 172 | H8 : mais oui pour des RMM. Commentaire : La plupart de mes réponses sont basées sur des sensations car je ne fais que passer dans les services pour voir les patients et les équipes médicales. Peu de lien avec les équipes soignantes. C'est pour cela aussi que beaucoup de réponses sont neutres | Evénements indésirables associés aux soins et coordination de la gestion du risque | RMM |
| 173 | Les classeurs sont-ils nécessaires dans les services alors qu'il existe "intranet" ? | Organisation | documents papier |
| 174 | Manque d'effectif qui nous épuise | Ressources humaines et soutien du management | Manque de personnel |
| 175 | Beaucoup de réponses neutres car tantôt d'accord ; tantôt pas d'accord selon les situations : difficile de faire une moyenne. | Questionnaire | difficultés |
| 176 | Questions stupides et pervers | Questionnaire | stupide |
| 177 | Questionnaire d'une grande subjectivité, il aurait été plus simple de faire une évalutation conjointe du stress des répondants. | Questionnaire | subjectif |
| 178 | L'UTEP ne reçoit pas de patients | Questionnaire | inadapté |
| 179 | Finalement je remplis beaucoup de questionnaires, mais les résultats ne sont pas toujours rendus auprès des soignants et autres corps de métiers. Je me pose la question si cela est pris en compte en espérant que cela améliore les situations. | Questionnaire | retour |
| 180 | Le questionnaire est bien fait. Je suis contente de l'avoir rempli. Nous aurons plus d'informations, par la voie hiérarchique, quannd tout le monde aura répondu au questionnaire. Merci encore pour cette enquête | Questionnaire | bien /retour |
| 181 | Les problèmes organisationnels sont travaillés pour être améliorés. Lorsque la sécurité des soins est remise en cause pour manque d'effectif, est-ce réellement le service qui est responsable ? | Ressources humaines et soutien du management | manque de personnel |
| 181 | Les problèmes organisationnels sont travaillés pour être améliorés. Lorsque la sécurité des soins est remise en cause pour manque d'effectif, est-ce réellement le service qui est responsable ? | Organisation | problèmes organisationnels |
| 182 | Le personnel soignant travaille souvent des conditions à flux tendu. Il n'est pas entendu par le personnel médical et la direction d'établissement et ça au détriment des patients. Ceci est fort regrettable. | Ressources humaines et soutien du management | flux tendu |
| 183 | Un service qui demande beaucoup de temps pour chaque résidents, de patience mais qui n'a pas assez de personnel fixe et un nombre insuffisant de personnels en journée / nuit. La sécurité est trop précaire et on ne nous donne pas assez de moyen pour prendre en charge les patients dans leur globalité. L'insécurité est trop présente, est stressante psychologiquement et physiquement. | Ressources humaines et soutien du management | manque de personnel /Stress /insécurité |
| 184 | Beaucoup de réponses neutes car nous avons une équipe fixe et peu de changement d'équipe. | Questionnaire | inadapté |
| 185 | A la question E j'ai répondu C par rapport à notre roulement en 12h qui peut engendrer des problèmes de sécurité quand nous faisons 13h ou que nous faisons plus de 48h / semaine = fatigue du personnel. | Organisation | horaisres / fatigue |
| 186 | Charge de travail trop importante par rapport aux personnels présents. Beaucoup de soins (transfusion, chimio…) je ne dis pas qu'il ne faut rien avoir à faire, le traviail on le fait mais quelquefois au détriment de l'accompagnement des personnes en fin de vie et des familles. Beaucoup trop de brancardage, de désorganisation (sorties des patients non prévues, entrées des patients non prévues). | Ressources humaines et soutien du management | Charge de travail |
| 186 | Charge de travail trop importante par rapport aux personnels présents. Beaucoup de soins (transfusion, chimio…) je ne dis pas qu'il ne faut rien avoir à faire, le traviail on le fait mais quelquefois au détriment de l'accompagnement des personnes en fin de vie et des familles. Beaucoup trop de brancardage, de désorganisation (sorties des patients non prévues, entrées des patients non prévues). | Organisation | manque /entrées / sorties |
| 187 | A17 : ça ne se produit pas ? | Questionnaire | précision |
| 188 | Je ne réponds pas aux questions catégories H, il enlève la confidentialité et l'anonymat par ses questions très précises. | Questionnaire | anonymat |
| 189 | De plus en plus de travail avec un effectif de personnel constant ! De plus en plus de patients dépendants avec les même moyens qu'il y a 8 ans = épuisement professionnel. | Ressources humaines et soutien du management | charge de travail / manque de personnel / épuisement |
| 190 | Le manque d'effectif accentue l'insécurité des soins. | Ressources humaines et soutien du management | manque de personnel |
| 191 | A7 : "Non concerné3 | Questionnaire | précision |
| 192 | Questionnaire plutôt ciblé personnel de soins infirmiers-médecins… Réponses difficiles pour les administratifs | Questionnaire | Questionnaire plutôt ciblé personnel de soins infirmiers-médecins |
| 193 | Pas adapté à notre profession | Questionnaire | non adapté |
| 194 | Avec l'augmentation importante des patients par jour, la sécurité des patients est mise à mal. Une grave erreur peut survenir à tout moment par le trop grand nombre de patients par IDE. | Ressources humaines et soutien du management | charge de travail / manque de personnel / fatigue |
| 195 | Pas de soutien des supérieurs hiérarchiques directs (cadre, cadre sup). Nous demandons une meilleure qualité des soins, plus de sécurité mais on ne nous en donne pas les moyens. Toujours moins de personnel et pas d'aide, de confiance de nos supérieurs. Continuité des soins égal qualité des soins égal équipe fixe. | Ressources humaines et soutien du management | manque de personnel |
| 196 | Manque d'effectif aide-soignant important et récurrent. Le service n'est pas dans les textes où il est dit qu'il doit y avoir 1 AS pour 4 patients. Sur l'horaire de nuit : 2 AS pour 18 malades aud lieu de 4 AS = mise en danger, mauvaise prise en charge, épuisement professionnel. | Ressources humaines et soutien du management | manque de personnel / épuisement |
| 197 | Revoir toute l'organisation des services (partage équitable des tâches). Privilégier la communication et le suivi patient et non le rendement. Unir les équipes entre elles | Ressources humaines et soutien du management | rendement |
| 197 | Revoir toute l'organisation des services (partage équitable des tâches). Privilégier la communication et le suivi patient et non le rendement. Unir les équipes entre elles | Organisation | répartition des taches / communication |
| 198 | Trop peu de personnel. Roulement trop dense donc surcharge de travail. Sécurité des soins à mon sens baffouée ! | Ressources humaines et soutien du management | manque de personnel / turnover / |
| 199 | Le manque de personnel nous fatigue de plus en plus. | Ressources humaines et soutien du management | manque de personnel /fatigue |
| 200 | Qestionnaire très mal conçu. Phrases mal faites, voire incompréhensibles. Les questions C et D exigent des réponses sous forme de fréquences (toujours, jamais, parfois...) alors que les réponses à cocher sont de l'ordre du ressenti !!! Apprenez à rédiger un questionnaire. qui a pondu ce truc !!! / Annotations particulière à : *nous vous remercions = vous pouvez ! ** enquête = nulle | questionnaire | critiques |
| 201 | Travailler en sous effectif --> sécurité des soins non assurée | Ressources humaines et soutien du management | manque de personnel |
| 202 | Pour assurer une meilleure sécurité des soins il faudrait plus de personnel, pour les patients et aussi plus de solidarité entre nous pour une meilleure entraide. | Ressources humaines et soutien du management | plus de personnel |
| 202 | Pour assurer une meilleure sécurité des soins il faudrait plus de personnel, pour les patients et aussi plus de solidarité entre nous pour une meilleure entraide. | Organisation | solidarité |
| 203 | H5 et H6 : 2-3 ans ==> que mettre ? | Questionnaire | précision |
| 204 | Le changement de fonctionnement du service provoque souvent des pertes d'informations au moment de l'entrée du patient, qui se font au détriment de l'équipe soignante et parfois du patient. | Organisation | perte d'information |
| 205 | Ce serait intéressant et utile que soit mis en place à La Bâtie des fiches de signalement d'évènements indésirables afin de tracer et lister les actions. | Evénements indésirables associés aux soins et coordination de la gestion du risque | Ne connait pas système de signalement |
| 206 | J'ai mis la réponse "neutre" lorsque la situation dépend de circonstances spéciales par exemple question 16 lorsque nous avons temporairement une surcharge de travail dans l'urgence ou autre | Questionnaire | précision |
| 207 | Manque de personnel : pas de pool, pas d'intérimaire, arrêt long non remplacé. Manque de considération de la charge de travail faite en plus du manque de personnel. Toutes les heures supplémentaires ne peuvent être récupérées ou ne sont pas payées, sachant que toutes ne sont pas notées par les soignants... Beaucoup à dire pour un si petit espace ! Question G : Réponse B, commentaire : pas plus car aucun retour. | Ressources humaines et soutien du management | manque de personnel / remplacements / heures supplémentaires |
| 207 | Manque de personnel : pas de pool, pas d'intérimaire, arrêt long non remplacé. Manque de considération de la charge de travail faite en plus du manque de personnel. Toutes les heures supplémentaires ne peuvent être récupérées ou ne sont pas payées, sachant que toutes ne sont pas notées par les soignants... Beaucoup à dire pour un si petit espace ! Question G : Réponse B, commentaire : pas plus car aucun retour. | Evénements indésirables associés aux soins et coordination de la gestion du risque | retour signalements |
| 208 | Trop de pression, d'informations. De plus en plus de patients, trop d'interruptions dans les soins est-ce sécurisant ? Malgré l'écoute et le soutien des responsables de l'unité et le travail en collaboration pour améliorer la prise en charge de la sécurité. | Organisation | interruptions / pression |
| 209 | En espérant que ce questionnaire soit utile à une amélioration en matière de sécurité !!! | Questionnaire | utilité ? |
| 210 | Qestionnaire pas fait pour les secrétaires. | Questionnaire | non adapté |
| 211 | Etant administrative je ne peux pas répondre à votre enquête | Questionnaire | non adapté |
| 212 | Les questions du type D ne peuvent pas être mises en lien avec les réponses du type : pas d'accord, d'accord, neutre... | Questionnaire | critique |
| 213 | Désolée pour le manque de prise d'opinion, mais je suis secrétaire et délocalisée de l'unité d'hospitalisation. Difficile de répondre aux questions sur le travail des équipes. Merci$ | Questionnaire | non adapté |
| 214 | A7 : "Ne s'évalue pas dans notre service". Certaines questions ne sont pas pertinentes. De plus, les situations changent en permanence, je trouve donc difficile de répondre à ces questions, et ne voit pas l'intérêt que cela m'apportera dans mon quotidien professionnel | Questionnaire | difficultés |
| 215 | Trop de travail, pas assez de personnel, pas assez d'écoute de la part des médecins du service. | Ressources humaines et soutien du management | manque de personnel / |
| 215 | Trop de travail, pas assez de personnel, pas assez d'écoute de la part des médecins du service. | Organisation | écoute des médecins |
| 216 | Au regard de la spécificité de fonctionnement de cette unité, j'ai dû répondre neutre sur des questions qui nous concernent moins dans un service d'hospitalisation clinique du CHU. L'US1 est une unité de consultation ambulatoire. | Questionnaire | non adapté |
| 217 | Ce questionnaire n'est pas anonyme pour tout le monde ! Nous sommes très peu de kiné, avec les infos sur le sexe et l'ancienneté… CE N'EST PLUS ANONYME! | Questionnaire | anonymat |
| 218 | Question A5 mal formulée | Questionnaire | critiques |
| 219 | Enquête intéressante | Questionnaire | intéressante |
| 220 | A quand un questionnaire sur la sécurité du personnel soignant. La direction de l'établissement montre que la qualité des produits n'est pas la priorité (cathéter IV…) donc la sécurité du personnel et la qualité des soins. | Ressources humaines et soutien du management | qualité non prioritaire |
| 220 | A quand un questionnaire sur la sécurité du personnel soignant. La direction de l'établissement montre que la qualité des produits n'est pas la priorité (cathéter IV…) donc la sécurité du personnel et la qualité des soins. | Environnement et matériel | qualité du matériel |
| 221 | Manque crucial de personnel dans le service (très forte necessité de créer des postes supplémentaires) par rapport à la charge de travail et à la spécificité du service. Aucune attractivité (notamment financière) pour attirer et garder de nouvelles personnes. | Ressources humaines et soutien du management | Manquede personnel / attractivité |
| 222 | Plus de personnel égal moins de risque d'erreurs. Plus de remplacement égal moins d'heures supplémentaires = moins de fatigue = moins d'erreurs. | Ressources humaines et soutien du management | Manque de personnel /remplacements / heures suplémentaires / Fatigue |
| 223 | C'est difficile pour ma part de répondre à ce questionnaire n'étant pas en ligne directe pour les soins médicaux proprements dits. | Questionnaire | non adapté |
| 224 | Nous travaillons dans des conditions qui sont à la limite de la sécurité du patient et du personnel. Question A5 cf les heures supplémentaires que nous faisons. Je n'ai pas eu le temps de faire la formation sécurité des soins sur intranet, 3 mois c'est trop juste comme délai, pas le temps de le faire sur le temps de travail. | Ressources humaines et soutien du management | manque de temps/ formation |
| 225 | La sécurité des soins est présente dans l'esprit de tous les soignants du service. Il est tout de même difficile de travailler en toute sécurité avec si peu de personnel pour autant de résidents. | Ressources humaines et soutien du management | manque de personnel |
| 226 | Point de vue intérimaire mais ayant éxpérience > 1an au | Questionnaire | précision |
| 227 | Dégradation de la sécurité des soins par manque de personnel et charge de travail trop importante évidemment. | Ressources humaines et soutien du management | manque de personnel |
| 228 | Effectif de soignants non pourvus en cas de pénurie (surtout AS). Très peu d'aide de la part d'autres services (pool peu présent quand manque de personnel). Architecture des locaux notamment les soins intensifs SIU non conventionnelle c'est-à-dire 2 chambres de 2 lits. Dans le cas de départ en examen, le patient côté porte doit être déplacé pour laisser passer son voisin côté fenêtre. Problème se pose pour le patient intubé/ventilé, qui est donc déplacé : risque d'extubation ou d'arrachage de VVC = sécurité des soins ? Ce n'est jamais arrivé car on travaille bien. | Ressources humaines et soutien du management | manque de personnel |
| 228 | Effectif de soignants non pourvus en cas de pénurie (surtout AS). Très peu d'aide de la part d'autres services (pool peu présent quand manque de personnel). Architecture des locaux notamment les soins intensifs SIU non conventionnelle c'est-à-dire 2 chambres de 2 lits. Dans le cas de départ en examen, le patient côté porte doit être déplacé pour laisser passer son voisin côté fenêtre. Problème se pose pour le patient intubé/ventilé, qui est donc déplacé : risque d'extubation ou d'arrachage de VVC = sécurité des soins ? Ce n'est jamais arrivé car on travaille bien. | Environnement et matériel | locaux |
| 229 | Certaines questions pas forcément adaptées à ma profession, donc difficulté pour répondre. | Questionnaire | non adapté |
| 230 | Question G : on a arrêté car on a l'impression qu'il n'y a pas de retour ! | Evénements indésirables associés aux soins et coordination de la gestion du risque | Pas de retour |
| 231 | Le problème principal dans notre service reste le nombre croissant de patients pour toujours le même nombre de personnel. | Ressources humaines et soutien du management | charge de travail |
| 232 | Pas adapté pour le personnel administratif, questionnaire pour les soignants. | Questionnaire | non adapté |
| 233 | En espérant que ce questionnaire serve à quelque chose et que nous ayons un retour, merci. | Questionnaire | utilité ? |
| 234 | Trop peu de personnel pour faire face à la charge de travail du service. Un passage en 12h serait certainement favorable à l'amélioration des conditions de travail. | Ressources humaines et soutien du management | manque de personnel / horaires |
| 235 | Rubrique D : ça ne veut rien dire | Questionnaire | critiques |
| 236 | Les sous effectifs sont la première cause d'erreurs car on veut gagner du temps, les choses sont faites trop vites. | Ressources humaines et soutien du management | manque de personnel / rapidité |
| 237 | Questionnaire difficilement remplissable du fait de ma profession administrative | Questionnaire | non adapté |
| 238 | Manque de personnel très important, il faut tout le temps s'adapter et faire tout en même temps, servir un repas&donner un TTT en même temps. Insécurité au niveau des prescriptions des internes ex lavenox & calciforme prescrit en même temps à 20h. insécurité : difficile de joindre les internes la nuit par exemple. Rattraper les mauvaises prescriptions ou les prescriptions non faites. Travail de nuit=solitude++ car médecin ne répond pas | Ressources humaines et soutien du management | Manque de personnel |
| 238 | Manque de personnel très important, il faut tout le temps s'adapter et faire tout en même temps, servir un repas&donner un TTT en même temps. Insécurité au niveau des prescriptions des internes ex lavenox & calciforme prescrit en même temps à 20h. insécurité : difficile de joindre les internes la nuit par exemple. Rattraper les mauvaises prescriptions ou les prescriptions non faites. Travail de nuit=solitude++ car médecin ne répond pas | Organisation | médecins difficiles à joindre |
| 239 | Manque de coordination entre les services au niveau des secrétariats médicaux (de nombreux dossiers se baladent et l'imagerie est restituée parfois 3 mois après la sortie du patient). | Organisation | coordination |
| 240 | Quand les brancardiers du bloc viennent chercher les patients, ils n'ont pas (et c'est régulier) le nom du patient à opérer. Et c'est idem pour nous, pour les entrées, les médecins ne nous communique pas toujours la venue du patient, ni son nom (pas très accueillant pour le patient) | Organisation | information / communication |
| 241 | Pas assez de personnel, de plus en plus de traçabilité et papiers qui alourdissent la prise en charge des soignants. Peu de temps pour beaucoup de soins et papiers. | Ressources humaines et soutien du management | manque de personnel |
| 241 | Pas assez de personnel, de plus en plus de traçabilité et papiers qui alourdissent la prise en charge des soignants. Peu de temps pour beaucoup de soins et papiers. | Organisation | travail adinistratif / traçabilité |
| 242 | Le personnel et les soignants souffrent énormément du manque de personnel soignant. Beaucoup trop de petits erreurs (qui heureusement n'ont jamais abouti en grosses erreurs) sont faites. Heureusement que pour certains la conscience professionnelle existe encore. Trop de travail, personne ne nous écoute : nous souffrons. | Ressources humaines et soutien du management | manque de personnel / souffrance |
| 243 | Faire un débat autour d'une table sur le questionnaire (plus intéressant° Merci | Questionnaire | retour |
| 244 | Un service de sécurité plus présent qui passe dans le service la nuit car personnel réduit +++ | Sécurité du personnel | service de sécurité plus présent |
| 245 | Pas très adapté à notre service… | Questionnaire | non adapté |
| 246 | Depuis 2 ans pas de remplacement d'une ASH à 100% | Ressources humaines et soutien du management | remplacement |
| 247 | Gros problème d'effectif pour les aides-soignantes. Ex : la nuit, pour la réa, 2 aides-soignantes pour 18 malades = équipe fatiguée, surmenée. La journée on court sans arrêt (pas de pause) pour pallier le manque d'effectif dans les unités pour assurer malgré tout les soins. C'est très bien de nous faire remplir le questionnaire et nous vous en remercions, mais serons-nous entendus et des actions seront-elles mises en place ? | Ressources humaines et soutien du management | manque de personnels |
| 247 | Gros problème d'effectif pour les aides-soignantes. Ex : la nuit, pour la réa, 2 aides-soignantes pour 18 malades = équipe fatiguée, surmenée. La journée on court sans arrêt (pas de pause) pour pallier le manque d'effectif dans les unités pour assurer malgré tout les soins. C'est très bien de nous faire remplir le questionnaire et nous vous en remercions, mais serons-nous entendus et des actions seront-elles mises en place ? | Questionnaire | utilité ? |
