# Supplementary material for: The contribution of open comments to understanding the results from the Hospital Survey on Patient Safety Culture (HSOPS): A qualitative study
Source: PLoS One. 2018 Apr 19;13(4):e0196089. doi: 10.1371/journal.pone.0196089 (PMC5908235; doi:10.1371/journal.pone.0196089)
Supplement: S2 Table — (DOCX) [file pone.0196089.s002.docx]

S2 Table – aggregated closed questions database

| Dimension - item | Missing value*, *n* (%) | | Mean (SD) | |
| --- | --- | --- | --- | --- |
| Overall perceptions of safety | 6 | 3.21 | 3.21 | (0.40) |
| A10r | 64 | 3.36 | 3.36 | (5.60) |
| A15 | 57 | 3.05 | 3.05 | (6.89) |
| A17r | 73 | 3.15 | 3.15 | (5.64) |
| A18 | 53 | 3.29 | 3.29 | (2.74) |
| Frequency of event reporting | 94 | 3.39 | 3.39 | (1.33) |
| D1 | 106 | 3.31 | 3.31 | (2.80) |
| D2 | 109 | 3.34 | 3.34 | (2.38) |
| D3 | 120 | 3.51 | 3.51 | (1.83) |
| Supervisor expectations & actions | 33 | 3.49 | 3.49 | (1.02) |
| B1 | 45 | 3.29 | 3.29 | (7.62) |
| B2 | 48 | 3.44 | 3.44 | (5.18) |
| B3r | 47 | 3.56 | 3.56 | (2.86) |
| B4r | 47 | 3.69 | 3.69 | (3.02) |
| Organizational learning | 7 | 3.4 | 3.4 | (0.21) |
| A6 | 49 | 3.53 | 3.53 | (2.06) |
| A9 | 63 | 3.35 | 3.35 | (3.14) |
| A13 | 90 | 3.32 | 3.32 | (2.42) |
| Teamwork within hospital units | 0 | 3.54 | 3.54 | (0.37) |
| A1 | 40 | 3.71 | 3.71 | (2.65) |
| A3 | 21 | 3.84 | 3.84 | (1.58) |
| A4 | 18 | 3.55 | 3.55 | (3.00) |
| A11 | 46 | 3.05 | 3.05 | (14.45) |
| Communication openness | 14 | 3.5 | 3.5 | (0.29) |
| C2 | 36 | 3.82 | 3.82 | (1.71) |
| C4 | 41 | 2.98 | 2.98 | (8.08) |
| C6r | 25 | 3.69 | 3.69 | (2.25) |
| Feedback and communication about error | 16 | 3.38 | 3.38 | (0.66) |
| C1 | 62 | 3.03 | 3.03 | (7.55) |
| C3 | 40 | 3.44 | 3.44 | (3.25) |
| C5 | 31 | 3.66 | 3.66 | (2.59) |
| Nonpunitive response to error | 7 | 2.94 | 2.94 | (1.10) |
| A8r | 57 | 2.91 | 2.91 | (7.13) |
| A12r | 40 | 2.91 | 2.91 | (9.69) |
| A16r | 82 | 3.01 | 3.01 | (4.36) |
| Staffing | 0 | 2.89 | 2.89 | (0.58) |
| A2 | 36 | 2.38 | 2.38 | (23.34) |
| A5r | 122 | 3.01 | 3.01 | (7.73) |
| A7r | 140 | 3.72 | 3.72 | (4.67) |
| A14r | 38 | 2.45 | 2.45 | (18.96) |
| Hospital management support | 48 | 2.67 | 2.67 | (2.62) |
| F1 | 86 | 2.51 | 2.51 | (14.78) |
| F8 | 104 | 2.93 | 2.93 | (8.19) |
| F9r | 91 | 2.57 | 2.57 | (11.85) |
| Teamwork across hospital units | 47 | 3.04 | 3.04 | (0.13) |
| F2r | 77 | 2.45 | 2.45 | (12.75) |
| F4 | 89 | 2.98 | 2.98 | (4.95) |
| F6r | 84 | 3.49 | 3.49 | (1.92) |
| F10 | 101 | 3.27 | 3.27 | (3.06) |
| Hospitals handoffs & transitions | 67 | 2.88 | 2.88 | (0.46) |
| F3r | 118 | 2.63 | 2.63 | (6.87) |
| F5r | 129 | 2.96 | 2.96 | (5.45) |
| F7r | 113 | 2.76 | 2.76 | (4.00) |
| F11r | 126 | 3.16 | 3.16 | (3.96) |
